# Trends and transition dynamics of heated tobacco product use in Japan, 2015–2025: a repeated cross-sectional and longitudinal study

**DOI:** 10.1016/j.lanwpc.2026.101990

**Published:** 2026-09-25

**Authors:** Satomi Odani, Takahiro Tabuchi

**Affiliations:** a Zatum LLC, Grand Blanc, MI, USA; b Tohoku University Graduate School of Medicine, Sendai, Japan

**Keywords:** Heated tobacco products, Tobacco use, Transition dynamics, Longitudinal study, Japan

## Abstract

**Background:**

Heated tobacco products (HTPs) diffused rapidly in Japan, but transition dynamics remain unclear. We examined trends in HTP use and routes underlying its growth.

**Methods:**

Using data from the Japan Society and New Tobacco Internet Survey, 2015–2025, we estimated weighted prevalence of HTP use (N = 161,583). Sex- and age-standardised prevalence was used to estimate segmented trends with a 2018 knot. Among respondents observed in consecutive years (93,540 pairs), we fitted a multinomial transition model across five states (Never, Non-current, Cigarette-only, HTP-only, and Dual use) and decomposed changes in standardised HTP prevalence into four categories: uptake from Never/Non-current states, switching from Cigarette-only to HTP, switching back to Cigarette-only, and exit to Non-current use.

**Findings:**

Weighted HTP prevalence increased from 0.1% to 14.9% during 2015–2025. Standardised prevalence rose sharply through 2018 (3.33 percentage points/year) and more gradually thereafter (0.92 percentage points/year). Transition probabilities showed post-2018 persistence of HTP-only/Dual use (67.9% and 57.0%, respectively). In the longitudinal decomposition, mean annual entry into HTP use increased from 2.16 percentage points before 2018 to 3.48 after 2018, while mean annual exit changed from −0.39 to −2.30. After 2018, entry from Non-current states increased markedly, whereas contributions from Never states were smaller and switching from Cigarette-only to HTP remained substantial.

**Interpretation:**

HTP use in Japan shifted around 2018 from rapid expansion to slower but sustained growth. Within the transition-eligible sample, later growth reflected multiple dynamics—including continued cigarette-to-HTP switching, persistence within HTP-using states, and increasing uptake from Non-current states—rather than cigarette substitution alone.

**Funding:**

Japan Society for the Promotion of Science KAKENHI (25H01079).

**Research in context**

**Evidence before this study**

Before undertaking this study, we searched PubMed and Google Scholar for studies on heated tobacco product (HTP) use in Japan and related transition patterns, from January 1, 2015 to June 6, 2026, without language restrictions. Search terms included combinations of “heated tobacco product”, “heat-not-burn”, “IQOS”, “Japan”, “prevalence”, “transition”, “switching”, “dual use”, and “cessation”. Existing evidence showed rapid early increases in HTP use in Japan, and some studies also reported concurrent declines in cigarette sales or smoking prevalence, with especially rapid uptake between 2015 and 2018. However, previous studies varied in design, outcome definitions, and analytic focus, and most concentrated on repeated cross-sectional prevalence or selected switching outcomes. Evidence on whether subsequent HTP growth in Japan reflected continued substitution away from cigarettes or broader diffusion beyond current smokers remained incomplete. Overall, the available evidence was heterogeneous and mostly observational, with important methodological limitations because many studies relied on repeated cross-sectional or internet-based survey data. We identified no previous study that jointly quantified long-term annual changes in HTP prevalence and the transition pathways underlying that growth over time, and we found no pooled meta-analysis of these pathways in Japan.

**Added value of this study**

Using data from the Japan Society and New Tobacco Internet Survey from 2015 to 2025, we combined repeated cross-sectional analyses with longitudinal transition modelling and Markov decomposition to examine both long-term trends in HTP use and the transition pathways underlying those trends. We identified a clear shift around 2018 from rapid early expansion to slower but sustained growth. Our findings show that later growth in HTP use was not explained solely by continued acceleration of cigarette-to-HTP switching. Instead, persistence within HTP-only and Dual use states remained high, and uptake from Non-current groups became increasingly important, whereas contributions from Never states were smaller. By distinguishing persistence within HTP-using states from entry and exit routes, this study adds a mechanistic perspective that was not available from prevalence trends alone.

**Implications of all the available evidence**

Taken together with existing evidence, our findings suggest that HTP growth in Japan should not be interpreted simply as substitution away from cigarettes. Surveillance and policy evaluation should consider not only switching among current smokers, but also uptake among Never and Non-current groups, persistence of Dual use, and incomplete transition away from cigarettes. Japan provides an important early case for the Western Pacific region, where regulatory approaches to novel tobacco products remain heterogeneous. Future research should examine how specific regulatory and market conditions shape these transition pathways and whether similar patterns emerge in other settings.

## Introduction

Heated tobacco products (HTPs) spread rapidly in Japan following the introduction of IQOS in 2014 and subsequent market expansion in 2015, making Japan one of the earliest and most dynamic HTP markets worldwide.^1^^,^^2^ During this period, cigarette sales declined substantially, and several studies also reported declines in smoking prevalence,^1^^,^^3^^,^^4^ prompting debate over whether HTPs primarily displaced combustible cigarettes or instead expanded the tobacco-use landscape. Japan also offers a distinctive policy setting in which to examine this question. HTPs were incorporated into the broader tobacco-control framework but were not treated identically to combustible cigarettes: they initially faced a lower tax burden,^5^^,^^6^ and use in designated heated-tobacco-only rooms was permitted under the revised Health Promotion Act,^7^^,^^8^ creating a regulatory environment that differed from that for conventional cigarettes.

Japan represents a critical early case of large-scale HTP diffusion in the Western Pacific region.^4^^,^^9^ Alongside other early HTP markets such as South Korea,^10^^,^^11^ and in contrast to more restrictive settings such as Australia,^12^ Japan provides an informative case for understanding how HTP uptake unfolds when HTPs are incorporated into tobacco-control frameworks but are regulated differently from combustible cigarettes, including with respect to taxation and smoke-free provisions. This regional context is relevant because regulatory approaches to novel tobacco products remain heterogeneous across the Western Pacific.^12^, ^13^, ^14^, ^15^, ^16^

Previous Japanese studies have documented rapid early HTP uptake: current HTP use increased from 0.2% in 2015 to 11.3% in 2019.^2^^,^^4^ Sales data showed a parallel market shift: the annual decline in per-capita cigarette sales accelerated from 1.5% during 2011–2015 to 10.5% during 2015–2018, while per-capita HTP sales increased by 149.0% annually during 2016–2018.^1^ After 2018, both trends slowed, with cigarette sales declining by 7.3% annually and HTP sales increasing by 8.1% annually through 2023.^1^ Previous research further showed that dual use remained common,^4^^,^^17^ and longitudinal evidence suggested that HTP use did not promote smoking cessation or prevent relapse.^18^^,^^19^ However, these studies have mainly described repeated cross-sectional prevalence trends or selected switching outcomes, and therefore do not show which transition pathways accounted for the continued growth of HTP use over time. In particular, it remains unclear whether increasing HTP prevalence in Japan has been sustained mainly by switching from cigarette-only use, by persistence within HTP-using states, or by uptake among never or non-current users. Clarifying the relative contribution of these pathways is important for interpreting whether HTP growth reflects substitution away from cigarettes or a broader diffusion process within the tobacco-use landscape.

This question has broader regional and international relevance. WHO has stated that HTPs are tobacco products and that the WHO Framework Convention on Tobacco Control applies to them, reinforcing the need to address HTPs within established tobacco-control frameworks.^20^ At the same time, HTPs are often discussed in terms of harm reduction or cigarette substitution, whereas WHO documents also emphasise prevention of initiation among non-smokers and youth and caution against unproven health claims.^20^, ^21^, ^22^ Clarifying whether growth in HTP use reflects substitution alone or a broader diffusion process is therefore important as countries in the Western Pacific and beyond determine how HTPs should be regulated within existing tobacco-control systems.

Using data from the Japan Society and New Tobacco Internet Survey (JASTIS), 2015–2025, we examined long-term trends in HTP use in Japan and quantified the transition pathways underlying its growth.

## Methods

### Study design and data source

We used annual data from the JASTIS, 2015–2025, an internet panel survey with annual replenishment, for repeated cross-sectional analyses of HTP prevalence and longitudinal analyses of 1-year transitions among respondents observed in consecutive years. All analyses were restricted to respondents aged 18–69 years; longitudinal transition pairs required age eligibility in both the origin and destination years. The cross-sectional analytic dataset included 161,583 annual observations from 58,751 unique respondents, and the longitudinal analyses included 93,540 consecutive-year transition pairs from 35,479 respondents. To characterise panel follow-up and potential attrition-related selection, we summarised annual retention, non-observation while remaining age-eligible, ageing out, return after a gap, first-time participation, total waves contributed, and maximum consecutive-wave participation. We also compared sex, age, and tobacco-use state across observations contributing valid next-year pairs, those not observed in the next wave while remaining age-eligible, and those that aged out. Cross-sectional analyses used the available year-specific cross-sectional weights to improve representativeness for each survey year. Detailed methodology of the JASTIS has been reported previously.^23^ Sampling procedures and survey administration remained consistent across waves; product-specific items were updated as the market changed, but the core recall periods and analytic definitions remained unchanged. Because appropriate longitudinal weights were unavailable for the replenished panel, longitudinal analyses were unweighted and interpreted as internally standardised dynamics among transition-eligible respondents rather than nationally representative transition rates. Sex and date of birth were self-reported at Rakuten Insight panel registration, with sex recorded as a binary variable. Gender identity was not collected and therefore could not be analysed. Race and ethnicity were not collected because these variables were not included in either the Rakuten Insight panel-registration data or the core JASTIS survey items used in this study. The JASTIS protocol was approved by the Research Ethics Committee of the Osaka International Cancer Institute (approval number 1611079163; approved November 7, 2016). All participants provided electronic informed consent.

### Tobacco-use states

In each survey year, respondents reported past-30-day use of each HTP product then marketed in Japan. The product list was updated annually (Supplementary Table S1), while the recall period and analytic definition remained unchanged. Current HTP use was defined as use of at least one listed HTP product on at least 1 day in the past 30 days. Combustible cigarettes and HTPs were separately classified as Never, Non-current (any lifetime use but no past-30-day use), or Current (use on at least 1 day in the past 30 days) and combined into five mutually exclusive states: Never, Non-current, Cigarette-only, HTP-only, and Dual use (Table 1). Current HTP use comprised HTP-only and Dual use. The Never state was cumulative: once a respondent had any history of combustible cigarette or HTP use, later observations could not return to Never. In this manuscript, “Non-current” denotes the five-state classification, whereas “former cigarette smokers” (any lifetime use but no past-30-day use) refers specifically to cigarette-smoking status. E-cigarette use was not incorporated into the state classification because nicotine-containing e-cigarettes were regulated as pharmaceutical products, no domestically approved nicotine-containing e-cigarette products were available, and survey ascertainment was complicated by confusion with HTPs.

**Table 1:** Definitions of product-specific use status and the five mutually exclusive tobacco-use states.

3-category status for combustible cigarettes and HTPs, assessed separately	
Product-specific status	Definition
Never	No lifetime use of the relevant product category
Non-current	Any lifetime use of the relevant product category, but no use in the past 30 days
Current	Use of the relevant product category on at least 1 day in the past 30 days

Five mutually exclusive tobacco-use states, based on combustible cigarette and HTP status	
State	Definition
Never	No lifetime use of combustible cigarettes or HTPs
Non-current	Any lifetime use of combustible cigarettes or HTPs, but no current use of either product
Cigarette-only	Current combustible cigarette use and no current HTP use
HTP-only	Current HTP use and no current combustible cigarette use
Dual use	Current use of both combustible cigarettes and HTPs

Product-specific use status was assessed separately for combustible cigarettes and HTPs. When applied to combustible cigarettes in the stratified cross-sectional analyses, Never use, Non-current use, and Current use corresponded to never, former, and current cigarette smokers, respectively. A former cigarette smoker could currently use HTPs and therefore could be classified as HTP-only in the five-state classification. In contrast, the five-state Non-current category required no current use of either combustible cigarettes or HTPs. Current HTP use, the primary outcome in the cross-sectional analyses, comprised HTP-only and Dual use; current combustible cigarette use comprised Cigarette-only and Dual use. HTP use was assessed using named products available in each survey wave, as listed in Supplementary Table S1.

### Cross-sectional prevalence and trend analyses

We estimated annual weighted prevalence of current HTP use overall and by sex, age group, and cigarette-smoking status; corresponding unweighted estimates and weighted prevalence of all five states were also calculated. To account for compositional change over time, we directly standardised annual prevalence to fixed pooled distributions from the analytic sample: sex-by-age for overall and smoking-status strata, age for sex-specific analyses, and sex for age-specific analyses. Annual percent change (APC) and annual absolute change (AAC) were estimated from segmented models fitted to the standardised prevalence series. Candidate 1-knot years were compared using the Bayesian information criterion (BIC) in weighted prevalence models adjusted for sex and age, with agreement between model-based and directly standardised prevalence as a secondary criterion; 2018 was selected as the common change point (Supplementary Table S2). APC was not reported for unstable segments, defined as fewer than 2 finite annual standardised prevalence values or a minimum standardised prevalence below 0.1%.

### Longitudinal transition analyses

Consecutive annual observations were paired to examine how respondents moved between the five tobacco-use states over 1 year; respondents observed in more than two consecutive waves could contribute multiple pairs. Because analyses were conditional on participation in both waves, selective panel retention was possible. We estimated the probability that respondents in each origin state would remain in the same state or move to another state in the following year using multinomial logistic regression, with destination state as the outcome and origin state, calendar time, sex, and age group as predictors. The main model included origin-state-by-time interactions to allow transition patterns to change over time. Candidate 1-knot years were compared using a parsimonious model and BIC; 2018 was selected and used for period-specific summaries (Supplementary Table S6). Model-based annual transition probabilities were standardised to the pooled sex-by-age distribution of the transition-eligible sample and summarised for 2016–2018 and 2019–2025.

### Markov decomposition of annual change in HTP prevalence

To identify which transition pathways contributed to annual growth in HTP prevalence, we used the estimated annual transition probabilities to reconstruct how the distribution of the five tobacco-use states changed from one year to the next, starting from 2015. We then divided each annual change in reconstructed HTP prevalence into four pathways: uptake from Never or Non-current states, switching from Cigarette-only to HTP use, switching back to Cigarette-only, and exit to Non-current use. We also summarised total entry into and exit from HTP use. Because the decomposition used the same transition-eligible sample and standardisation basis as the transition model, it represents internally standardised longitudinal change rather than directly decomposing weighted national prevalence.

### Uncertainty and sensitivity analyses

For weighted and unweighted prevalence estimates, transition probabilities, and decomposition summaries, 95% confidence intervals were estimated using respondent-level cluster bootstrap resampling. As a structural sensitivity analysis, we compared the main model with a simpler model without origin-state-by-time interactions using BIC and agreement between reconstructed and observed aligned HTP prevalence. To assess the potential influence of repeated survey participation, additional analyses adjusted for time in sample, defined as the cumulative number of JASTIS waves contributed up to and including the origin wave and categorised as 1, 2, 3, 4, or ≥5. To assess sensitivity to potentially greater short-term variability in tobacco-use behaviours and lower next-wave retention among younger adults, we repeated the longitudinal transition and decomposition analyses after restricting pairs to respondents aged 25–69 years in both the origin and destination years. Further details are provided in the Supplementary Material.

### Role of the funding source

The funder had no role in study design, data collection, data analysis, data interpretation, writing of the report, or the decision to submit the manuscript for publication.

## Results

### Cross-sectional prevalence and trend analyses

Weighted prevalence of current HTP use increased markedly over the study period, from 0.1% in 2015 to 14.9% in 2025 (Fig. 1; Supplementary Table S3). It rose steeply to 7.5% by 2018 and then more gradually to 10.6% in 2019 and 14.9% in 2025. Throughout the study period, weighted prevalence of current HTP use was consistently higher among men than women (22.2% vs. 7.7% in 2025) and increased in all age groups. In 2025, it ranged from 14.7% to 18.0% among adults aged 18–54 years and was 9.4% among those aged 55–69 years. Weighted prevalence of current HTP use was highest among current cigarette smokers, increasing from 0.6% in 2015 to 43.7% in 2025. Corresponding prevalence increased from 0.1% to 18.4% among former cigarette smokers, whereas it remained substantially lower among never cigarette smokers (0.0%–1.4%). Unweighted prevalence of current HTP use showed a similar overall pattern, increasing from 0.2% in 2015 to 14.4% in 2025, although weighting affected the magnitude of estimates in some years and strata (Supplementary Table S4).

**Fig. 1:**
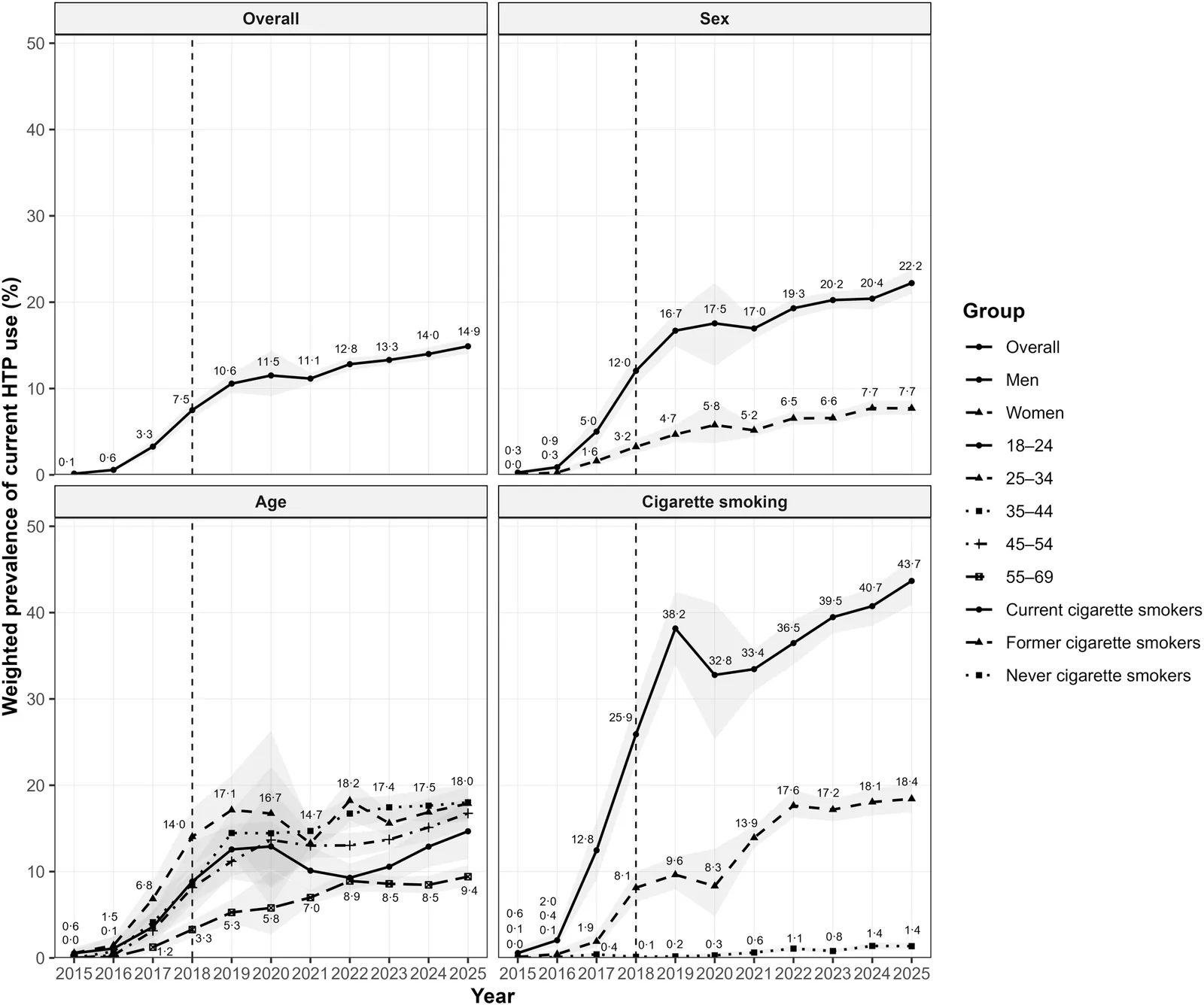
Weighted prevalence of current heated tobacco product (HTP) use in Japan, 2015–2025, overall and by sex, age group, and cigarette-smoking status. Current HTP use comprised HTP-only and Dual use and was based on use on at least 1 day in the past 30 days. Shaded areas indicate 95% cluster bootstrap confidence intervals. The dashed vertical line marks 2018, the selected common change point used in segmented analyses. For age groups, numeric labels are shown only for the highest and lowest prevalence in each year.

Weighted prevalence of HTP-only and Dual use increased from 0.0% and 0.1% in 2015 to 6.5% and 8.4% in 2025, respectively, with Dual use remaining more prevalent throughout (Table 2). Over the same period, Cigarette-only use decreased from 19.9% to 10.9%, while Non-current use increased from 22.1% to 28.1%.

**Table 2:** Weighted prevalence of five mutually exclusive tobacco-use states in Japan, 2015–2025.

Year	Never% (95% CI)	Non-current% (95% CI)	Cigarette-only% (95% CI)	HTP-only% (95% CI)	Dual use% (95% CI)
2015	57.8 (55.6, 59.7)	22.1 (20.4, 23.9)	19.9 (18.3, 21.8)	0.0 (0.0, 0.1)	0.1 (0.0, 0.2)
2016	56.2 (53.5, 58.6)	23.5 (21.2, 26.0)	19.7 (17.9, 21.9)	0.2 (0.1, 0.3)	0.4 (0.3, 0.6)
2017	54.4 (51.8, 56.7)	24.1 (21.9, 26.6)	18.2 (16.0, 20.5)	0.7 (0.5, 0.9)	2.6 (1.9, 3.4)
2018	51.1 (49.3, 53.0)	26.7 (25.1, 28.3)	14.6 (13.4, 16.0)	2.4 (1.9, 3.0)	5.1 (4.3, 5.9)
2019	48.4 (46.5, 50.4)	28.9 (26.8, 31.0)	12.1 (10.9, 13.3)	3.1 (2.5, 3.7)	7.5 (6.5, 8.5)
2020	42.2 (38.4, 45.8)	28.0 (24.5, 31.1)	18.3 (15.3, 21.9)	2.6 (1.5, 3.9)	8.9 (6.8, 11.6)
2021	50.3 (49.2, 51.6)	24.8 (23.7, 25.9)	13.7 (12.9, 14.6)	4.3 (3.9, 4.6)	6.9 (6.3, 7.5)
2022	50.4 (49.2, 51.6)	24.0 (23.1, 25.0)	12.8 (12.0, 13.5)	5.5 (5.0, 6.0)	7.3 (6.8, 7.9)
2023	49.3 (48.4, 50.2)	25.1 (24.4, 25.9)	12.3 (11.6, 12.8)	5.3 (4.9, 5.7)	8.0 (7.5, 8.5)
2024	48.0 (47.0, 49.0)	26.7 (25.8, 27.5)	11.4 (10.8, 12.0)	6.2 (5.6, 6.7)	7.8 (7.3, 8.5)
2025	46.1 (44.9, 47.2)	28.1 (27.1, 29.2)	10.9 (10.3, 11.6)	6.5 (5.9, 7.0)	8.4 (7.8, 9.2)

Values are weighted percentages with 95% confidence intervals in parentheses. Tobacco-use states were mutually exclusive and were defined using lifetime history and past 30-day use of combustible cigarettes and heated tobacco products (HTPs). HTP-only indicates current HTP use without current combustible cigarette use, and Dual use indicates current use of both HTPs and combustible cigarettes. Estimates were calculated using year-specific cross-sectional weights, and 95% confidence intervals were estimated using respondent-level cluster bootstrap resampling.

### Segmented trends in standardised prevalence of current HTP use

Model-comparison results supported 2018 as the common change point (Supplementary Table S2). Overall standardised prevalence of current HTP use increased rapidly during 2015–2018 (APC, 283.4% per year; 95% CI, 215.5–340.8; AAC, 3.33 percentage points per year; 95% CI, 2.92–3.72) and more slowly during 2019–2025 (APC, 5.7%; 95% CI, 3.0–8.3; AAC, 0.92 percentage points per year; 95% CI, 0.74–1.11) (Table 3). Before 2018, absolute annual increases in standardised prevalence of current HTP use were larger among men than women (AAC, 5.21 vs. 1.50 percentage points/year) and were especially pronounced among current and former cigarette smokers (10.55 and 3.44 percentage points/year, respectively). After 2018, standardised prevalence of current HTP use continued to increase among current, former, and never cigarette smokers (AAC, 1.83, 1.43, and 0.15 percentage points/year, respectively) and across age groups (range, 0.57–1.20 percentage points/year).

**Table 3:** Segmented trends in the prevalence of current heated tobacco product (HTP) use in Japan: annual percent change (APC) and annual absolute change (AAC) for 2015–2018 and 2019–2025.

	2015–2018		2019–2025	
	APC, %/year (95% CI)	AAC, pp/year (95% CI)	APC, %/year (95% CI)	AAC, pp/year (95% CI)
Overall	283.4 (215.5, 340.8)	3.33 (2.92, 3.72)	5.7 (3.0, 8.3)	0.92 (0.74, 1.11)
Sex				
Men	267.6 (204.9, 372.7)	5.21 (4.51, 5.83)	5.3 (2.2, 8.1)	1.27 (0.96, 1.58)
Women	–	1.50 (1.14, 1.90)	6.0 (0.9, 10.5)	0.58 (0.40, 0.75)
Age				
18–24	177.7 (119.7, 304.3)	3.63 (2.71, 4.72)	4.1 (−1.2, 10.0)	0.59 (0.01, 1.10)
25–34	194.7 (132.4, 331.7)	5.06 (3.90, 6.37)	1.7 (−3.5, 6.2)	0.57 (0.03, 1.10)
35–44	–	4.17 (3.22, 5.20)	6.5 (3.5, 9.4)	1.20 (0.78, 1.62)
45–54	–	3.47 (2.68, 4.33)	7.2 (4.0, 9.6)	1.13 (0.72, 1.53)
55–69	–	1.56 (1.15, 2.08)	12.1 (−11.0, 18.6)	0.88 (0.65, 1.08)
Cigarette smoking				
Never	–	0.05 (−0.02, 0.13)	25.0 (−2.1, 36.3)	0.15 (0.10, 0.20)
Former	185.9 (115.1, 332.1)	3.44 (2.67, 4.15)	10.9 (2.6, 16.4)	1.43 (1.09, 1.79)
Current	242.4 (176.1, 355.9)	10.55 (9.16, 11.83)	2.9 (−0.1, 5.8)	1.83 (1.27, 2.39)

APC is presented as % per year and AAC as percentage points (pp) per year; both are shown with 95% confidence intervals. Trend estimates were based on sex- and age-standardised prevalence series using 2018 as the selected common change point. For the pre-2018 segment, annual changes were estimated over 2015–2018, with 2015 serving as the baseline year. A dash (–) indicates that APC was not estimated for unstable segments, defined as fewer than 2 finite annual standardised prevalence values or a minimum standardised prevalence below 0.1%.

### Panel follow-up and transition-eligible sample

Previous-wave retention ranged from 62.5% to 75.6% across the 2016–2025 waves; annual samples also included first-time participants and respondents returning after missed waves (Supplementary Table S5, Panel A). Across origin years, 2111 observations aged out before the subsequent wave. Compared with observations not followed while remaining age-eligible, pair-contributing observations more often came from men (51.7% vs. 46.6%), fewer adults aged 18–24 years (8.4% vs. 20.6%), and more aged 55–69 years (30.4% vs. 19.6%); tobacco-use-state distributions were broadly similar (Supplementary Table S5, Panel B). Of 58,751 unique respondents, 37,880 (64.5%) contributed at least two waves and 9547 (16.2%) contributed five or more, although these waves were not necessarily consecutive (Supplementary Table S5, Panel C). Consecutive-wave participation attenuated more substantially: 35,479 (60.4%) contributed at least two consecutive waves, 22,657 (38.6%) at least three, 14,250 (24.3%) at least four, and 8264 (14.1%) at least five (Supplementary Table S5, Panel D).

### Longitudinal transition probabilities

Selected model-based, sex- and age-standardised 1-year transition probabilities within the transition-eligible sample are shown in Fig. 2; full annual 5 × 5 transition probabilities and two-period summary matrices are provided in Supplementary Table S7 and Fig. S1. Among Cigarette-only users, persistence decreased from 76.8% in 2016–2018 to 64.4% in 2019–2025, while transition to Non-current use increased from 13.5% to 22.0%. Transitions to HTP-only remained low (2.1% vs. 2.7%), and transitions to Dual use increased modestly (7.6% vs. 11.0%); both cigarette-to-HTP pathways rose around 2018 but did not continue to accelerate. Transitions from Never to HTP use remained infrequent, increasing from 0.6% in 2016–2018 to 1.3% in 2019–2025 (Supplementary Table S7).

**Fig. 2:**
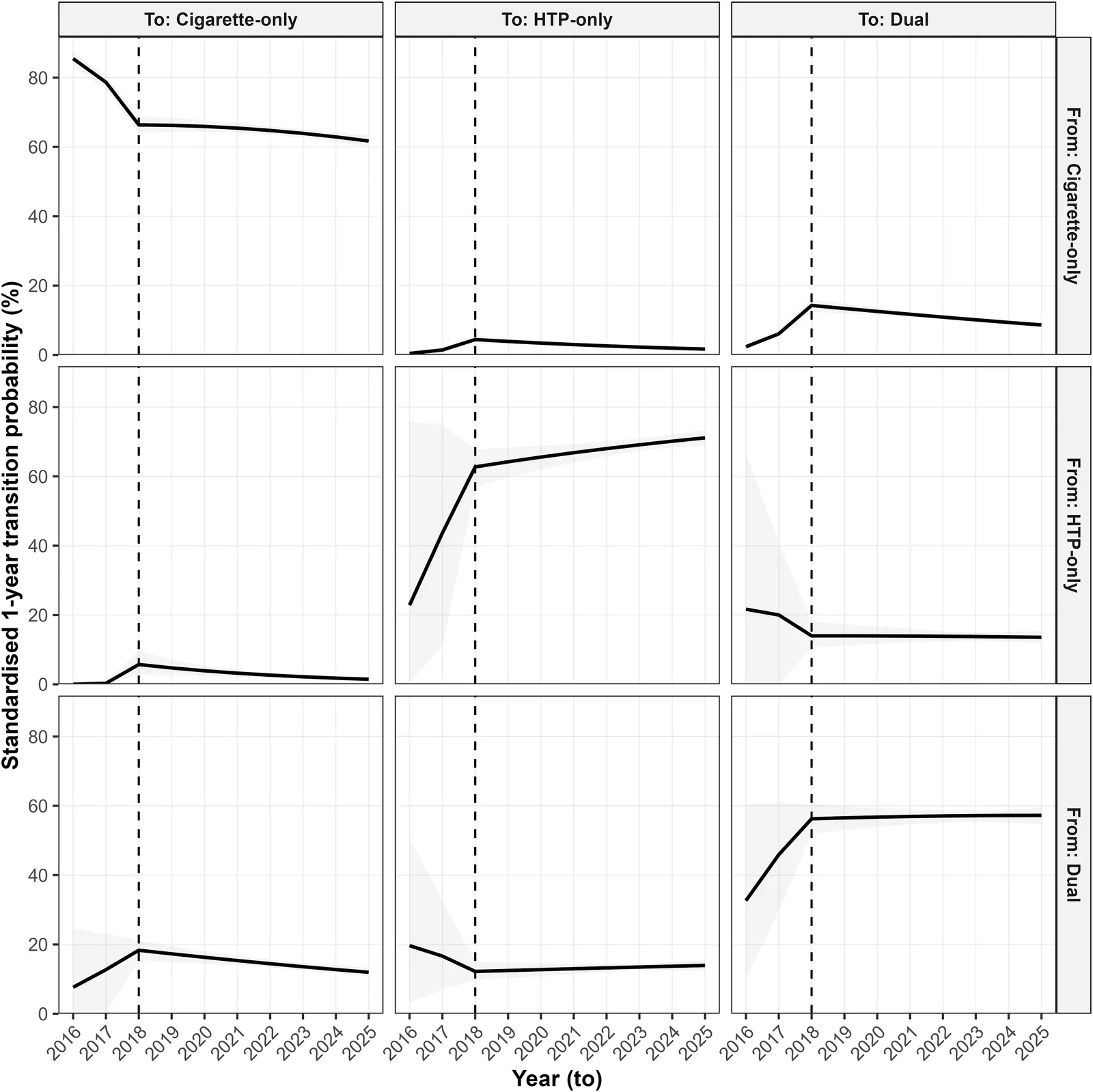
Selected model-based, sex- and age-standardised 1-year transition probabilities among Cigarette-only, HTP-only, and Dual use states in Japan, 2016–2025. Rows indicate origin states and columns indicate destination states. Selected panels show transitions between Cigarette-only, HTP-only, and Dual use states; full five-state transition probabilities are provided in Supplementary Fig. S1. Shaded areas indicate 95% cluster bootstrap confidence intervals. The dashed vertical line marks 2018, the selected common change point used in period-specific summaries.

Persistence in HTP-using states remained high after 2018. The probability of remaining HTP-only increased from 43.1% to 67.9%, while remaining Dual increased from 44.9% to 57.0%. Corresponding transitions to Non-current use decreased from 36.4% to 15.5% among HTP-only users and from 26.0% to 15.3% among Dual users. Transitions between HTP-only and Dual use remained common in both directions (13.2%–18.6%). Estimates for HTP-only users in 2016–2018 were less precise.

### Markov decomposition of annual change in HTP prevalence

The annual net change in reconstructed HTP prevalence remained positive throughout 2016–2025 and peaked in 2018 at 2.96 percentage points (Fig. 3). In period-specific summaries, entry contributions outweighed exit contributions in both periods, indicating a positive net change in HTP prevalence within the aligned transition-eligible sample. Fig. 3 presents the four-category decomposition; all ten transition routes are shown in Supplementary Table S8 and Fig. S2.

**Fig. 3:**
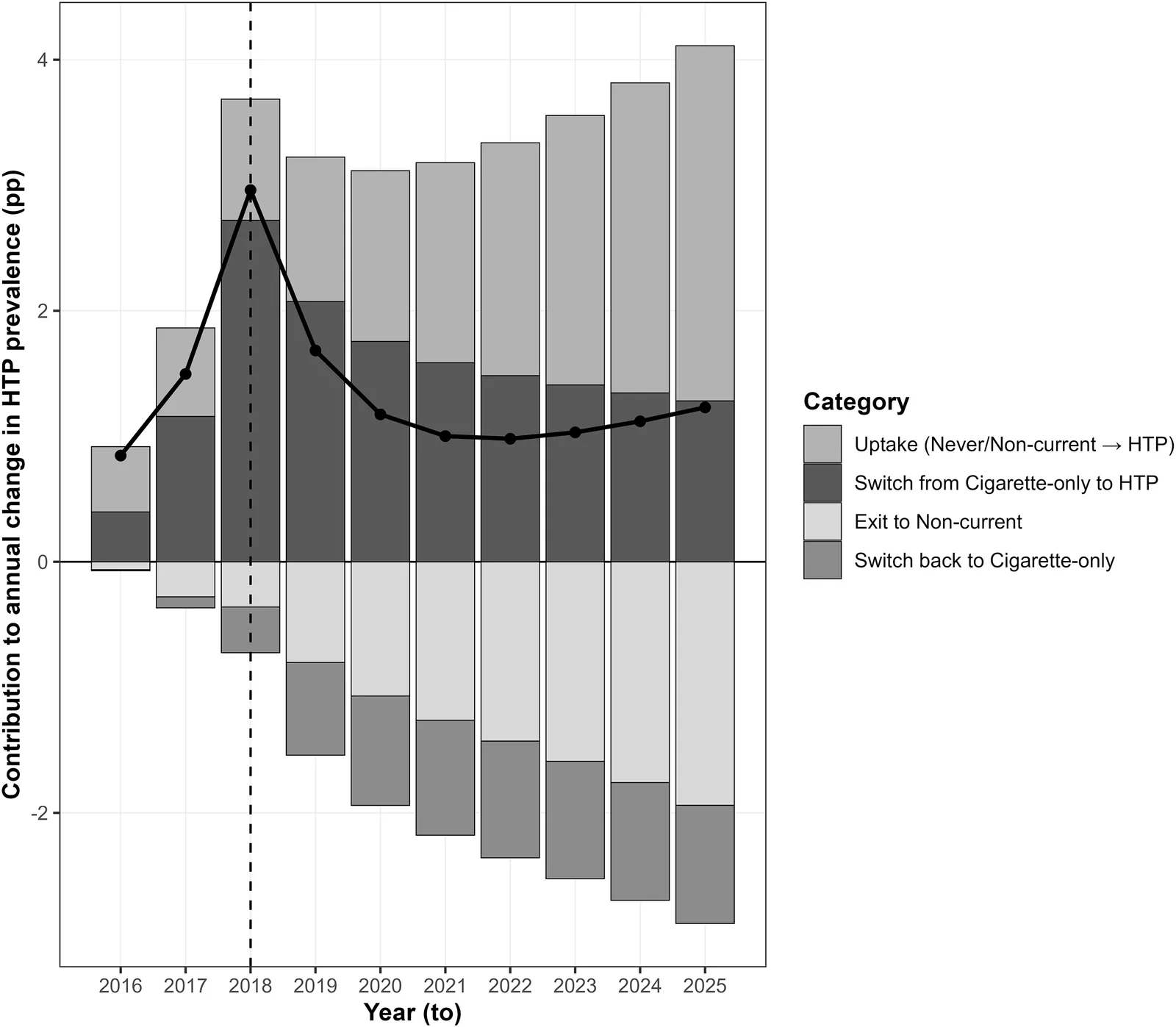
Markov decomposition of annual change in HTP prevalence in Japan, 2016–2025. Bars show annual contributions from four route categories: uptake from Never or Non-current use, switching from Cigarette-only to HTP use, switching back to Cigarette-only, and exit to Non-current use. The black line indicates the annual net change in reconstructed HTP prevalence. Positive values indicate contributions to increasing HTP prevalence and negative values indicate contributions to decreasing HTP prevalence. The dashed vertical line marks 2018.

During 2016–2018, switching from Cigarette-only to HTP use contributed more to increasing prevalence than uptake from Never/Non-current states (mean annual contributions, 1.43 vs. 0.73 percentage points; slopes, 1.16 vs. 0.22 percentage points/year). During 2019–2025, the mean annual contribution from Cigarette-only-to-HTP switching remained substantial at 1.56 percentage points but declined over time (slope, −0.12 percentage points/year). By contrast, mean annual contributions from Never and Non-current states increased from 0.39 and 0.34 percentage points during 2016–2018 to 0.52 and 1.40 percentage points during 2019–2025, respectively. Thus, although transitions from Never to HTP use were infrequent at the individual level, they continued to make a positive contribution to reconstructed annual changes in HTP prevalence, while the larger post-2018 increase in uptake was driven primarily by Non-current states (Supplementary Table S8).

Negative contributions also increased in magnitude after 2018: switching back to Cigarette-only changed from −0.15 to −0.90 percentage points, and exit to Non-current use from −0.23 to −1.41 percentage points.

### Sensitivity analyses

Sensitivity analyses supported the robustness of the main findings. The main transition model showed better fit and slightly better reconstruction of aligned HTP prevalence than the simpler model without origin-state-by-time interactions, although the simpler model yielded broadly similar transition and decomposition results (Supplementary Tables S9–S11). Additional adjustment for time in sample and restriction of the analyses to respondents aged 25–69 years also produced materially similar findings: during 2019–2025, uptake from Never/Non-current states continued to increase, whereas the contribution from Cigarette-only-to-HTP switching declined over time (Supplementary Tables S12 and S13).

## Discussion

In this study, current HTP use in Japan increased markedly between 2015 and 2025, with a clear shift around 2018 from rapid early expansion to slower but sustained growth. Although prevalence remained highest among men and current cigarette smokers, increases were also observed among former cigarette smokers, whereas HTP prevalence among never cigarette smokers remained substantially lower (1.4% in 2025). Longitudinal analyses showed that, after 2018, persistence in HTP-only and Dual use remained high, whereas the probability of transitioning from Cigarette-only to HTP-only use no longer increased. Decomposition analyses showed that Cigarette-only-to-HTP transitions contributed substantially before 2018, whereas uptake from Non-current states became more important thereafter. Transitions from Never to HTP use were infrequent at the individual level, but nevertheless made a positive contribution to annual changes in reconstructed HTP prevalence within the transition-eligible sample. The annual net change in reconstructed HTP prevalence remained positive throughout. The cross-sectional and longitudinal findings were qualitatively consistent: rapid weighted prevalence growth before 2018 coincided with increasing contributions from Cigarette-only-to-HTP transitions, whereas slower growth thereafter coincided with declining contributions from this pathway and larger exits from HTP use. Overall, later HTP growth reflected not only switching from cigarettes but also persistence in HTP-using states and uptake beyond current smokers.

These findings caution against interpreting HTP diffusion solely as substitution for combustible cigarettes. HTPs are frequently framed by industry and in some policy discussions as substitutes for combustible cigarettes.^21^^,^^22^^,^^24^ However, our findings indicate that cigarette-to-HTP switching contributed substantially to the initial expansion of HTP use, but that later growth was sustained through multiple dynamics, including continued use within HTP-only and Dual states and increasing uptake from Non-current states. Current HTP use among former cigarette smokers may likewise reflect both complete switching from cigarettes and HTP uptake after cigarette cessation. Persistent Dual use further indicates that HTP uptake does not invariably lead to complete displacement of combustible cigarettes. These findings do not negate concurrent declines in cigarette smoking or complete switching among some smokers; rather, they show that the observed expansion of HTP use cannot be understood from cigarette substitution alone. Even where complete switching may reduce exposure for individual smokers, the broader public health implications of HTP diffusion also depend on uptake among Never and Non-current groups and the persistence of Dual use. HTPs should therefore be evaluated both as potential substitutes for cigarettes and as products diffusing more broadly within the tobacco-use landscape.

Our findings support previous Japanese evidence of rapid HTP uptake, persistent dual use, and concurrent early declines in cigarette use.^1^^,^^2^^,^^4^^,^^18^ The slower but sustained post-2018 increase in HTP prevalence is also consistent with market-level evidence that growth in HTP sales and declines in cigarette sales both moderated after the initial expansion.^1^^,^^3^, ^4^, ^5^, ^6^, ^7^, ^8^ By combining repeated cross-sectional trends with longitudinal transition modelling and Markov decomposition, this study extends that literature by showing that the pathways sustaining HTP growth changed over time.

These temporal and transition patterns emerged amid changes in tobacco taxation, revised smoke-free regulations, and maturation of the HTP market around and after 2018.^1^^,^^3^, ^4^, ^5^, ^6^, ^7^, ^8^ Narrowing price differences and differential treatment of HTPs under smoke-free regulations may have affected the relative appeal of HTPs and cigarettes, but these changes coincided with evolving product availability, marketing, and consumer familiarity. The analyses therefore describe HTP diffusion under Japan’s policy and market conditions rather than identify the effect of any single policy.

These findings have broader policy implications. Prevention and surveillance should encompass HTP uptake among Never and Non-current groups, while cessation services should address HTP-only and Dual use alongside combustible cigarette smoking. HTP diffusion should not be treated as a substitute for comprehensive tobacco-control and cessation measures; regulatory assessment should consider population-level outcomes—including uptake among nonusers, dual use, and incomplete switching—rather than infer benefit from cigarette displacement alone. For Western Pacific countries considering HTP regulation, taxation, and monitoring, Japan’s experience suggests that declining cigarette use does not necessarily reflect simple or uniformly beneficial substitution.^18^^,^^24^ The U.S. regulatory treatment of IQOS illustrates a related point: although FDA authorised IQOS for marketing and granted reduced-exposure MRTP orders for certain products, its framework does not treat these products simply as cigarette substitutes and explicitly considers population-level effects, including uptake among nonusers, dual use, and complete switching.^25^, ^26^, ^27^ This perspective is consistent with our findings and may also be relevant beyond the region, particularly in high-income markets where HTPs or similar products are expanding.^14^^,^^16^

This study has several strengths. It used a long observation window spanning 2015 to 2025, covering the early introduction, rapid expansion, and later maturation of the HTP market in Japan. It also combined repeated cross-sectional and longitudinal approaches, allowing assessment of both aggregate prevalence trends and the transition dynamics underlying those trends. In addition, sex- and age-standardisation reduced the influence of compositional changes over time, and the decomposition framework provided a structured way to quantify the contributions of different transition routes to changes in HTP prevalence.

Several limitations should be considered. First, JASTIS is an internet panel survey and may therefore be subject to selection bias. Directly comparable nationally representative estimates using the same population denominator, past-30-day definition, and age range were unavailable; greater emphasis should therefore be placed on temporal patterns than on exact national prevalence levels. Current HTP use was defined as use on at least 1 day in the past 30 days and may include occasional or experimental use. Second, longitudinal analyses were unweighted and conditional on consecutive-wave participation, and retained respondents differed in age and sex from those not observed in the next wave; the findings therefore reflect internally standardised dynamics within the transition-eligible sample rather than nationally representative longitudinal processes. Third, the cross-sectional former-cigarette-smoker category was not directly equivalent to the longitudinal Non-current state and did not distinguish cessation duration or incorporate prior nicotine dependence. Fourth, the longitudinal analyses examined 1-year transitions and could not characterise complete multi-year trajectories, longer-term cessation or relapse, or whether Dual use was temporary or persistent. The sample attenuated substantially with sustained follow-up: 38.6%, 24.3%, and 14.1% of respondents contributed at least three, four, and five consecutive waves, respectively; longer-term analyses would therefore involve progressively smaller and more selected samples. Finally, decomposition confidence intervals did not incorporate sampling variability in the baseline aligned state distribution, tobacco use was self-reported, and the analyses could not distinguish birth-cohort from age and period effects or causally attribute the shift around 2018 to any single policy, market, or social factor.

### Conclusion

HTP use in Japan shifted around 2018 from rapid expansion to slower but sustained growth. Cigarette-to-HTP switching remained an important pathway, while persistence within HTP-using states and increasing uptake from Non-current states also characterised later growth. Surveillance and policy evaluation should therefore consider switching among current smokers, persistence of Dual use, and uptake from Non-current and Never states. Future research should examine longer-term individual trajectories and how regulatory and market conditions shape these transitions in Japan and other settings.

## Contributors

SO: conceptualisation, methodology, formal analysis, visualisation, and writing—original draft. TT: conceptualisation, investigation, methodology, project administration, data curation, and writing—review and editing. SO and TT contributed to data interpretation and directly accessed and verified the underlying individual-participant data reported in the manuscript. Both authors had full access to all the data in the study, approved the final version of the manuscript, and accepted responsibility for the decision to submit the manuscript for publication. TT had final responsibility for the decision to submit.

## Data sharing statement

Deidentified individual participant data, a data dictionary, and the study protocol will be made available to researchers on reasonable request for academic research purposes. Data and related documents will be available from the time of publication, with no predetermined end date. Requests should be directed to the corresponding author, Takahiro Tabuchi (tabuchitak@gmail.com). Access will be subject to review of a methodologically sound proposal and, where applicable, completion of a data access agreement.

## Declaration of interests

SO is affiliated with Zatum LLC as an Epidemiologist and has received consulting fees from Zatum LLC and FMF Healthcare Research within the past 36 months. These relationships were unrelated to the present work. TT reports receiving research funding from Daiichi Sankyo Healthcare Co., Ltd., Johnson & Johnson K.K., Data Seed Inc., Workout-Plus LLC, EMMA Co., Ltd., Addness Inc., CotoIT Inc., and Laugh Inc. within the past 36 months. He also serves as a research advisor to Data Seed Inc. and receives personal consulting fees. These relationships were unrelated to the present work. None of the organisations listed above is related to the tobacco or nicotine industries.
