# Supplementary material for: Trends and transition dynamics of heated tobacco product use in Japan, 2015–2025: a repeated cross-sectional and longitudinal study
Source: Lancet Reg Health West Pac. 2026 Sep 25;75:101990. doi: 10.1016/j.lanwpc.2026.101990 (PMC13634990; doi:10.1016/j.lanwpc.2026.101990)
Supplement: Supplementary file 1 [file mmc1.pdf]

SUPPLEMENTARY MATERIAL

TABLE OF CONTENTS

SUPPLEMENTARY METHODS S1
Overall analytic framework S1
Assessment of HTP use S1
Table S1. HTP products assessed by survey year S1
Definition of tobacco-use states S2
Cross-sectional prevalence estimation S2
Direct standardisation of annual prevalence S3
Selection of the cross-sectional change point S3
APC and AAC from standardised prevalence series S3
Construction of 1-year transition data S4
Selection of the longitudinal change point S4
Multinomial transition models S5
Standardisation of annual transition matrices S5
Uncertainty in transition probabilities S5
Period-average transition probabilities S5
Markov decomposition of annual changes in HTP prevalence S6
Decomposition closure and period summaries S7
Uncertainty in decomposition results S7
Sensitivity analyses S7
Interpretation of estimands, missing data, and implementation S8
SUPPLEMENTARY RESULTS S9
S1. Cross-sectional analyses S9
Table S2. Cross-sectional candidate knot years S9
Table S3. Weighted prevalence of current HTP use S10
Table S4. Unweighted prevalence of current HTP use S11
S2. Longitudinal analyses S12
Table S5. Panel retention and survey participation history S13
Table S6. Longitudinal candidate knot years S16
Table S7. Transition probabilities: main model S16
Figure S1. Annual five-state transition probabilities S17
Table S8. Markov decomposition: main model S18
Figure S2. Annual route-specific contributions S19

S3. Sensitivity analyses S20
Table S9. Main versus simpler model diagnostics S21
Table S10. Transition probabilities: simpler model S21
Table S11. Markov decomposition: simpler model S22
Table S12. Markov decomposition adjusted for time in sample S22
Table S13. Markov decomposition among respondents aged 25–69 years S23

SUPPLEMENTARY METHODS

Overall analytic framework

The analyses consisted of two linked components. First, we estimated annual prevalence of current heated tobacco product (HTP) use using repeated cross-sectional analyses with year-specific cross-sectional weights. Second, we modeled 1-year transitions between mutually exclusive tobacco-use states among respondents observed in consecutive years and decomposed annual changes in HTP prevalence into contributions from specific transition routes. For the longitudinal component, the main analysis used a multinomial transition model that allowed time trends to differ by origin state (from×time interaction model). A simpler model assuming common time trends across origin states was evaluated as a structural sensitivity analysis. All analyses were restricted to respondents aged 18–69 years. Longitudinal transition pairs required respondents to be aged 18–69 years in both the origin and destination years.

Assessment of HTP use

In each survey year, respondents reported past-30-day use of each HTP product then marketed in Japan. The list of named products was updated annually to reflect changes in the Japanese market, while the recall period and analytic definition of current HTP use remained unchanged (Table S1). Current HTP use was defined as use of at least one listed HTP product on at least 1 day in the past 30 days.

Table S1. Heated tobacco products assessed in JASTIS by survey year, 2015–2025

Survey year	Product-specific HTP items
2015	Ploom; IQOS
2016	Ploom; IQOS
2017	Ploom TECH; IQOS; glo
2018	Ploom TECH; IQOS; glo
2019	Ploom TECH; Ploom TECH+; Ploom S; IQOS; glo
2020	Ploom TECH; Ploom TECH+; Ploom S; IQOS; glo; glo sens; PULZE
2021	Ploom TECH; Ploom TECH+; Ploom S; IQOS; glo; glo sens; glo hyper; PULZE
2022	Ploom TECH; Ploom S; Ploom X; IQOS; glo; lil HYBRID
2023	Ploom TECH; Ploom S; Ploom X; IQOS; glo; lil HYBRID
2024	Ploom TECH; Ploom X; IQOS; glo; lil HYBRID; with
2025	Ploom TECH; Ploom X; IQOS; glo; lil HYBRID; with

Each product was assessed using a separate questionnaire item asking about use during the past 30 days. The list of named products was updated annually to reflect products marketed in Japan; these updates represented changes in the product market rather than changes in the recall period or analytic definition of current HTP use. Product names

are presented as shown in the survey questionnaire. HTP, heated tobacco product; JASTIS, Japan Society and New Tobacco Internet Survey.

Definition of tobacco-use states

Combustible cigarettes and HTPs were separately classified as Never, Non-current, or Current based on lifetime history and past-30-day use. Non-current use was defined as any lifetime use of the relevant product category but no use in the past 30 days, and Current use as use in the past 30 days. These product-specific classifications were then combined into five mutually exclusive states: Never, Non-current, Cigarette-only, HTP-only, and Dual use. Current HTP use comprised HTP-only and Dual use; thus, the binary outcome in the cross-sectional analyses compared current HTP use with all other states. The Never state was treated as cumulative. Once a respondent had any history of combustible cigarette or HTP use, later observations were not allowed to return to Never. In practice, if a respondent with prior use history gave a later response corresponding to Never, that observation was recoded as Non-current rather than returned to Never. Therefore, transitions from any non-Never state to Never were structurally disallowed in the analytic state variable. Throughout the manuscript, "Non-current" denotes the five-state tobacco-use classification, whereas "former cigarette smokers" refers specifically to cigarette-smoking status and was defined as any lifetime combustible cigarette use but no use in the past 30 days. A former cigarette smoker could therefore be classified as HTP-only if currently using HTPs.
E-cigarette use was not incorporated into the state classification because nicotine-containing e-cigarettes were regulated as pharmaceutical products during the study period, no domestically approved nicotine-containing e-cigarette products were available, and survey ascertainment was complicated by confusion between e-cigarettes and HTPs.

Cross-sectional prevalence estimation

Annual weighted, unstandardised prevalence of current HTP use was estimated using the year-specific cross-sectional weight. Estimates were obtained for the overall sample and stratified by sex, age group, and cigarette-smoking status. Let w_{iy} denote the year-specific cross-sectional weight for respondent i in year y, and let $I(HTP_{iy} = 1)$ be an indicator of current HTP use. The weighted annual prevalence was estimated as

$$p_y = \frac{\sum_i w_{iy} \, I(HTP_{iy} = 1)}{\sum_i w_{iy}}.$$

Uncertainty was quantified using respondent-level cluster bootstrap resampling. In each bootstrap iteration, respondents were resampled with replacement, and the multiplicity with which a respondent was selected was applied to that respondent's annual weights across all observed years before recomputing weighted prevalence. Percentile bootstrap 95% confidence intervals (CIs) were obtained from the 2.5th and 97.5th percentiles of the bootstrap distribution. For comparison, corresponding unweighted prevalence estimates were calculated without

applying survey weights, using the same outcome definition, strata, and respondent-level cluster bootstrap framework.

Direct standardisation of annual prevalence

To reduce the influence of changing demographic composition over time, annual weighted prevalence was directly standardised to fixed pooled reference distributions derived from the analytic sample over 2015–2025. For the overall analysis and smoking-status-stratified analyses, we used a pooled sex-by-age reference distribution. For sex-stratified analyses, we used a pooled age distribution; for age-stratified analyses, we used a pooled sex distribution. Let z denote an adjustment stratum and q_z the fixed pooled reference weight for stratum z. If p_{yz} is the weighted prevalence in year y within stratum z, the directly standardised annual prevalence was

$$p_y^{std} = \sum_z q_z \; p_{yz}.$$

This approach allowed annual comparisons to be made against a common demographic structure and improved comparability across years and strata.

Selection of the cross-sectional change point

To identify a potential change point in the increasing trend of HTP use, we compared candidate one-knot segmented models in the overall sample using weighted sex- and age-adjusted prevalence models. Candidate knot years were restricted pragmatically to years that left approximately at least two observed years on each side of the knot. Model fit was compared primarily using the Bayesian information criterion (BIC). As a secondary check, we examined agreement between directly standardised observed prevalence and model-based standardised predicted prevalence, summarized using root mean squared error (RMSE). Based on these comparisons, 2018 was selected as the common change point for the segmented analyses.

APC and AAC from standardised prevalence series

Segmented trends in the standardised prevalence series were summarized using annual percent change (APC) and annual absolute change (AAC), separately for the periods before and after 2018. For year y, with 2015 as the reference year, we defined

$$t_y = y - 2015, h_y = (y - 2018)_+ = \max(0, y - 2018).$$

APC was obtained from segmented log-linear models fitted to the directly standardised annual prevalence series:

$$\log(p_y^{std}) = \alpha + \beta_1 t_y + \beta_2 h_y.$$

Thus,

$$APC_{pre} = 100\{\exp(\beta_1) - 1\},$$

and

$$APC_{post} = 100\{\exp(\beta_1 + \beta_2) - 1\}.$$

AAC was obtained from segmented linear models on the percentage-point scale:

$$p_y^{std} = \alpha + \gamma_1 t_y + \gamma_2 h_y.$$

Thus,

$$AAC_{pre} = \gamma_1,$$

and

$$AAC_{post} = \gamma_1 + \gamma_2.$$

APC was not reported for a given segment when fewer than two finite annual standardised prevalence values were available in that segment or when the minimum standardised prevalence in the segment was below 0.1%, because the log-scale estimate was considered unstable.

Uncertainty in APC and AAC was evaluated using the same respondent-level cluster bootstrap framework used for annual prevalence estimation. In each bootstrap iteration, weighted prevalence was recalculated within each year and standardization stratum, directly standardised again using the fixed reference distribution, and APC and AAC were recomputed from the resulting annual standardised prevalence series. Thus, both point estimates and CIs were based on the same directly standardised series. Analyses were conducted using R version 4.5.1, primarily with the stats, dplyr, tidyr, tibble, ggplot2, nnet, purrr, and rlang packages.

Construction of 1-year transition data

For the longitudinal analyses, 1-year transition records were constructed from person-year data. Observations for each respondent were ordered by year, and pairs of observations separated by exactly one year were linked, with the state in year y−1 treated as the origin state and the state in year y as the destination state. As a result, the longitudinal analyses were restricted to respondents observed in two consecutive years, and the resulting transition sample did not coincide with the annual cross-sectional samples. Because appropriate longitudinal weights were unavailable for this replenished panel, all longitudinal analyses were conducted without weighting and were interpreted as estimating panel-internal transition dynamics within the transition-eligible sample.

Selection of the longitudinal change point

To assess whether the change point supported in the cross-sectional prevalence analyses was also supported in the longitudinal component, candidate one-knot years were compared using a parsimonious multinomial logistic transition model without from-by-time interaction terms. The outcome was destination state, and predictors included origin state, calendar time, a hinge term defined by each candidate knot year, sex, and age group. Candidate years were again restricted to those leaving approximately two observed transition years on each side of the knot. Model fit was compared primarily using BIC. For this comparison, candidate knot years ranged from 2017 to 2023. Based on these comparisons, 2018 was selected as the best-supported knot. The main longitudinal analyses therefore used

2018 as the common change point, while allowing state-specific time trends through from-by-time interaction terms.

Multinomial transition models

We used multinomial logistic regression with destination state as the outcome and origin state, time, sex, and age group as predictors. In the main model, time trends were allowed to differ by origin state through from×time interaction terms. Using the segmented time variables defined above,

$$t_y = y - 2015, h_y = (y - 2018)_+,$$

the main model was

$$to \sim from + t_y + h_y + sex + age5 + from{:}\, t_y + from{:}\, h_y.$$

The simpler sensitivity model used the same segmented time structure but omitted the from×time interaction terms, thereby imposing common time trends across origin states:

$$to \sim from + t_y + h_y + sex + age5.$$

Standardisation of annual transition matrices

Because the sex and age composition of the transition-eligible sample could vary across years, model-predicted transition probabilities were standardised to the pooled sex-by-age distribution of the overall transition-eligible sample. Let z denote a sex-by-age stratum, q_z the proportion of stratum z in the pooled transition-eligible sample, and $\hat{P}_{y,rc}(z)$ the model-predicted probability of transition from origin state r to destination state c in year y within stratum z. The standardised transition probability was

$$\hat{P}^{std}_{y,rc} = \sum_{z} q_z \ \hat{P}_{y,rc}(z).$$

Collecting these cell-specific probabilities yielded the standardised transition matrix P^{std}_y for year y. These standardised matrices formed the basis of both the transition results and the decomposition analyses.

Uncertainty in transition probabilities

Uncertainty in annual standardised transition probabilities was evaluated using respondent-level cluster bootstrap resampling. In each iteration, respondents were resampled with replacement, all transition records from selected respondents were carried forward together, and duplicated respondents contributed duplicated transition series. The multinomial model was then re-estimated and the standardised transition matrix recalculated. Cell-specific 95% CIs were obtained from the percentile bootstrap distribution for each origin-state × year × destination-state combination.

Period-average transition probabilities

To facilitate interpretation, annual standardised transition probabilities were summarized over two periods: 2016–

2018 and 2019–2025. For each origin-to-destination cell, we calculated the mean transition probability within each period and the difference between post and pre periods. Percentile bootstrap 95% CIs were derived for each summary measure.

Markov decomposition of annual changes in HTP prevalence

To decompose annual changes in HTP prevalence into contributions from specific transition routes, we first defined an aligned observed state distribution consistent with the same target population and standardization basis as the transition model. For 2016–2025, this aligned distribution used the destination-state distribution among respondents contributing a valid one-year transition into each year. For baseline 2015, it used the origin-state distribution among respondents contributing transitions into 2016. These observed state distributions were directly standardised to the same pooled sex-by-age reference distribution used for the transition model, and the standardised 2015 state distribution served as the initial distribution for the decomposition.

Let π_y denote the standardised state distribution vector in year y. Using the year-specific standardised transition matrices, annual state distributions were recursively reconstructed in a discrete-time Markov framework:

$$\pi_y = \pi_{y-1} \mathrm{P}_y^{std}.$$

Reconstructed HTP prevalence in year y was defined as the sum of the HTP-only and Dual use proportions in the reconstructed state distribution:

$$Prev_y^{HTP} = \pi_{y,HTP\text{-}only} + \pi_{y,Dual}.$$

The year-to-year change in reconstructed HTP prevalence was then decomposed into route-specific contributions according to whether a transition moved respondents into or out of HTP-using states. Let $I_H(s)$ be an indicator taking value 1 if state s includes HTP use (HTP-only or Dual use) and 0 otherwise. The standardised flow from state r to state c in year y was

$$F_{y,rc} = \pi_{y-1,r} \hat{P}_{y,rc}^{std},$$

and the contribution of that transition to the annual change in HTP prevalence, expressed in percentage points, was

$$C_{y,rc} = 100\, F_{y,rc}\{I_H(c) - I_H(r)\}.$$

Thus, transitions from non-HTP states to HTP states contributed positively, transitions from HTP states to non-HTP states contributed negatively, and transitions that did not change HTP status contributed zero.

We examined ten specific transition routes: Never→HTP-only, Never→Dual, Non-current→HTP-only, Non-current→Dual, Cigarette-only→HTP-only, Cigarette-only→Dual, HTP-only→Cigarette-only, Dual→Cigarette-only, HTP-only→Non-current, and Dual→Non-current. These were further grouped into four categories: Uptake (Never/Non-current → HTP), Switch from cigarette → HTP, Switch back to cigarette, and Exit to Non-current. We also defined two broader aggregate indicators: Entry into HTP, equal to Uptake plus Switch from cigarette → HTP, and Exit from HTP, equal to Switch back to cigarette plus Exit to Non-current.

Decomposition closure and period summaries

By construction, the decomposition satisfied the closure property that the annual change in reconstructed HTP prevalence equaled the sum of the route-specific contributions:

$$\Delta Prev_y^{HTP} = Prev_y^{HTP} - Prev_{y-1}^{HTP} = \sum_{r,c} C_{y,rc}.$$

In implementation, we checked the residual difference between these two quantities as a closure diagnostic. Contributions were summarized for each individual route, each four-category aggregate, and each two-category aggregate for the periods 2016–2018 and 2019–2025. Summary measures included mean annual contribution, period-summed contribution, and the slope of annual contributions within each period, as well as the post–pre difference.

Uncertainty in decomposition results

Uncertainty in decomposition results was propagated from the bootstrap distribution of the standardised transition matrices. For each bootstrap iteration, annual transition matrices were recalculated, state distributions were recursively reconstructed, route-specific and category-specific annual contributions were recomputed, and period-summary measures were recalculated. Percentile bootstrap 95% CIs were then obtained for each summary measure. In the implemented analyses, however, the baseline aligned state distribution for 2015 was held fixed rather than re-estimated within each bootstrap iteration. Therefore, the reported CIs reflect uncertainty in the transition matrices but do not incorporate sampling variability in the baseline aligned distribution; for quantities close to the initial years, this may lead to somewhat narrower CIs than would be obtained under full uncertainty propagation.

Sensitivity analyses

As a structural sensitivity analysis, we fitted a simpler multinomial transition model without from×time interaction terms and repeated the same standardisation, Markov reconstruction, and decomposition procedures. The main model and the simpler model were compared using two criteria: BIC and agreement between reconstructed HTP prevalence and observed aligned HTP prevalence. The latter was assessed using year-specific differences in percentage points, mean absolute difference, maximum absolute difference, RMSE for the full state distribution, and mean absolute deviation (MAD). The from×time model showed better agreement with the aligned observed HTP prevalence series and more favorable information criteria and was therefore retained as the main model. For more aggregated indicators, especially Entry versus Exit, conclusions were also checked for robustness under the simpler model.

In two additional sensitivity analyses, we assessed the potential influence of time in sample and the inclusion of younger adults. First, time in sample was defined as the cumulative number of JASTIS survey waves contributed by each respondent up to and including the origin wave of each transition pair and was categorised as 1, 2, 3, 4, or ≥5

waves. This variable was added as a covariate to the main multinomial transition model, while retaining the 2018 segmented time structure and from×time interaction terms. Model-predicted transition probabilities were averaged over the pooled distribution of time in sample within each sex-by-age stratum and then standardised to the pooled sex-by-age distribution of the transition-eligible sample. Second, we repeated the longitudinal transition and decomposition analyses after restricting transition pairs to respondents aged 25–69 years in both the origin and destination waves. For this analysis, model-predicted transition probabilities were standardised to the pooled sex-by-age distribution of the age-restricted transition-eligible sample. The 2018 change point, Markov reconstruction, decomposition, and bootstrap procedures were otherwise unchanged in both analyses. Apart from the age restriction, the original model specification was retained in the age-restricted analysis.

Interpretation of estimands, missing data, and implementation

Because the target estimands differed between the cross-sectional and longitudinal components, interpretation was separated accordingly. The cross-sectional prevalence and trend analyses were weighted and describe annual prevalence patterns in the survey target population within the limits of the available weighting. In contrast, the longitudinal transition and decomposition analyses were unweighted but standardised estimates restricted to respondents observed in two consecutive years and therefore reflect transition dynamics in a transition-eligible panel sample with fixed sex-by-age composition rather than nationally representative annual transition rates.

Each analysis was restricted to observations with non-missing values for variables required by that model. Sex, age, and the combustible cigarette and HTP use variables used to define tobacco-use states were derived from mandatory survey items and were therefore complete in the analytic dataset. Smoking-status-stratified cross-sectional analyses used observations with available values for the stratifying variable. All CIs were calculated using percentile bootstrap methods. Analyses were implemented primarily using the R packages dplyr, tidyr, tibble, ggplot2, nnet, purrr, and rlang.

SUPPLEMENTARY RESULTS

S1. Cross-sectional analyses

S1.1 Knot selection in cross-sectional analysis

Candidate one-knot years from 2017 to 2023 were compared in weighted sex- and age-adjusted prevalence models. 2018 provided the best fit, with the lowest BIC and the smallest RMSE between directly standardised observed prevalence and model-based standardised predicted prevalence (Table S2). Relative to 2018, ΔBIC ranged from 97 for 2019 to 1035 for 2023, and ΔRMSE ranged from 0·001 to 0·016. All alternative knot years showed worse fit than 2018 according to both BIC and RMSE. These results supported selection of 2018 as the common change point for the cross-sectional segmented trend analyses.

S1.2 Detailed prevalence of HTP use

Detailed annual weighted prevalence estimates of current HTP use overall and by sex, age group, and cigarette-smoking status are shown in Table S3. Overall weighted prevalence increased from 0·1% in 2015 to 14·9% in 2025. Prevalence was consistently higher among men than women and highest among current smokers, while remaining lowest among adults aged 55 years or older. By 2025, weighted prevalence was 22·2% among men, 7·7% among women, 43·7% among current smokers, 18·4% among former smokers, and 1·4% among never smokers. Confidence intervals were wider in some early years and lower-prevalence strata. Corresponding unweighted estimates showed a broadly similar temporal pattern to the weighted estimates, although the magnitude differed in some years and strata (Table S4). Overall unweighted prevalence of current HTP use increased from 0·2% in 2015 to 14·4% in 2025.

Table S2. Candidate knot years for cross-sectional segmented trend analyses

Knot year	BIC	RMSE of standardised prevalence	ΔBIC	ΔRMSE	Selected
2018	95,485	0·008	0	0·000	Yes
2019	95,582	0·009	97	0·001	No
2017	95,702	0·016	217	0·008	No
2020	95,864	0·015	380	0·007	No
2021	96,162	0·019	677	0·012	No
2022	96,322	0·021	837	0·014	No
2023	96,519	0·023	1,035	0·016	No

Lower BIC and RMSE indicate better fit. ΔBIC and ΔRMSE are shown relative to the best-fitting model (knot year 2018).

Table S3. Weighted prevalence of current heated tobacco product use in Japan, 2015–2025, overall and by sex, age group, and cigarette-smoking status

	2015	2016	2017	2018	2019	2020	2021	2022	2023	2024	2025
	Prevalence % (95% CI)	Prevalence % (95% CI)	Prevalence % (95% CI)	Prevalence % (95% CI)	Prevalence % (95% CI)	Prevalence % (95% CI)	Prevalence % (95% CI)	Prevalence % (95% CI)	Prevalence % (95% CI)	Prevalence % (95% CI)	Prevalence % (95% CI)
Overall	0·1 (0·1, 0·3)	0·6 (0·4, 0·8)	3·3 (2·6, 4·0)	7·5 (6·5, 8·4)	10·6 (9·5, 11·7)	11·5 (9·1, 14·4)	11·1 (10·5, 11·8)	12·8 (12·1, 13·6)	13·3 (12·8, 13·9)	14·0 (13·3, 14·8)	14·9 (14·1, 15·7)
Sex											
Men	0·3 (0·1, 0·5)	0·9 (0·5, 1·3)	5·0 (3·6, 6·7)	12·0 (10·4, 14·0)	16·7 (14·9, 18·7)	17·5 (12·6, 22·2)	17 (15·8, 18·0)	19·3 (18·2, 20·4)	20·2 (19·3, 21·2)	20·4 (19·2, 21·6)	22·2 (21·0, 23·8)
Women	0·0 (0·0, 0·0)	0·3 (0·1, 0·5)	1·6 (1·1, 2·3)	3·2 (2·5, 4·1)	4·7 (3·8, 5·8)	5·8 (3·7, 8·5)	5·2 (4·4, 5·8)	6·5 (5·8, 7·3)	6·6 (5·9, 7·3)	7·7 (7·0, 8·6)	7·7 (7·0, 8·6)
Age, years											
18–24	0·5 (0·2, 1·0)	1·1 (0·2, 2·4)	3·6 (2·2, 5·3)	8·8 (5·0, 13·4)	12·6 (9·5, 15·5)	12·9 (10·4, 15·8)	10·1 (7·6, 12·9)	9·3 (7·9, 10·9)	10·6 (8·7, 12·4)	12·9 (10·5, 15·4)	14·7 (11·5, 18·1)
25–34	0·6 (0·1, 1·2)	1·5 (0·7, 2·5)	6·8 (4·9, 8·9)	14·0 (10·7, 17·3)	17·1 (13·5, 21·1)	16·7 (8·1, 26·3)	13·3 (11·4, 15·2)	18·2 (16·0, 20·7)	15·6 (14·1, 17·0)	16·9 (15·2, 18·6)	17·9 (15·9, 19·8)
35–44	0·0 (0·0, 0·0)	0·7 (0·3, 1·4)	4·1 (2·3, 7·1)	8·6 (6·9, 10·6)	14·5 (11·9, 17·0)	14·4 (6·1, 22·1)	14·7 (12·9, 16·4)	16·7 (15·0, 18·5)	17·4 (16·0, 18·8)	17·6 (15·8, 19·5)	18·0 (16·1, 20·1)
45–54	0·0 (0·0, 0·0)	0·3 (0·0, 0·7)	3·1 (1·5, 5·5)	8·1 (6·4, 10·0)	11·2 (9·1, 13·4)	13·7 (8·4, 19·6)	13 (11·7, 14·4)	13·0 (11·6, 14·5)	13·7 (12·5, 14·9)	15·1 (13·6, 16·7)	16·7 (15·1, 18·6)
55–69	0·0 (0·0, 0·1)	0·1 (0·0, 0·2)	1·2 (0·6, 2·2)	3·3 (2·4, 4·3)	5·3 (4·0, 6·8)	5·8 (2·7, 10·0)	7·0 (6·2, 7·8)	8·9 (7·7, 10·2)	8·6 (7·6, 9·4)	8·5 (7·5, 9·5)	9·4 (8·2, 10·8)
Cigarette smoking											
Never	0·0 (0·0, 0·0)	0·1 (0·0, 0·2)	0·4 (0·2, 0·6)	0·1 (0·0, 0·2)	0·2 (0·1, 0·3)	0·3 (0·1, 0·6)	0·6 (0·4, 0·9)	1·1 (0·8, 1·4)	0·8 (0·6, 1·0)	1·4 (1·0, 1·8)	1·4 (1·0, 1·8)
Former	0·1 (0·0, 0·3)	0·4 (0·1, 0·9)	1·9 (1·2, 2·9)	8·1 (6·5, 10·0)	9·6 (8·0, 11·7)	8·3 (4·8, 12·6)	13·9 (12·7, 15·3)	17·6 (16·3, 19·4)	17·2 (15·8, 18·4)	18·1 (16·5, 19·6)	18·4 (16·9, 20·2)
Current	0·6 (0·1, 1·2)	2·0 (1·3, 3·0)	12·5 (9·1, 15·9)	25·9 (22·6, 29·7)	38·2 (33·9, 42·4)	32·8 (25·3, 41·1)	33·4 (30·9, 35·8)	36·5 (34·0, 39·2)	39·5 (37·6, 41·3)	40·7 (38·5, 42·8)	43·7 (40·8, 46·1)

Values are weighted percentages with 95% confidence intervals in parentheses. Current HTP use was defined as use of at least one HTP product on at least 1 day in the past 30 days and comprised HTP-only and Dual use. Estimates were calculated using year-specific cross-sectional weights. Confidence intervals were estimated using respondent-level cluster bootstrap resampling. CI, confidence interval; HTP, heated tobacco product.

Table S4. Unweighted prevalence of current heated tobacco product use in Japan, 2015–2025, overall and by sex, age group, and cigarette-smoking status

	2015	2016	2017	2018	2019	2020	2021	2022	2023	2024	2025
	Prevalence % (95% CI)	Prevalence % (95% CI)	Prevalence % (95% CI)	Prevalence % (95% CI)	Prevalence % (95% CI)	Prevalence % (95% CI)	Prevalence % (95% CI)	Prevalence % (95% CI)	Prevalence % (95% CI)	Prevalence % (95% CI)	Prevalence % (95% CI)
Overall	0·2 (0·1, 0·3)	0·8 (0·6, 1·1)	3·5 (3·1, 4·0)	7·5 (6·9, 8·0)	9·5 (9·0, 10·1)	8·6 (8·0, 9·3)	11·4 (11·0, 11·9)	11·8 (11·5, 12·3)	12·3 (11·9, 12·7)	13·2 (12·8, 13·6)	14·4 (14·0, 14·9)
Sex											
Men	0·3 (0·2, 0·5)	1·1 (0·8, 1·5)	4·7 (3·8, 5·5)	10·8 (9·9, 11·7)	14·1 (13·1, 15·2)	11·8 (10·8, 12·8)	16·9 (16·2, 17·6)	17·4 (16·8, 18·1)	18·3 (17·6, 19·0)	19·1 (18·3, 19·7)	20·6 (19·8, 21·3)
Women	0·1 (0·0, 0·2)	0·5 (0·2, 0·8)	2·4 (1·8, 3·0)	3·9 (3·4, 4·5)	5·0 (4·4, 5·7)	4·9 (4·2, 5·6)	5·8 (5·3, 6·2)	6·4 (6·1, 6·9)	6·5 (6·1, 6·9)	7·5 (7·0, 8·0)	8·4 (7·8, 8·9)
Age, years											
18–24	0·8 (0·3, 1·4)	2·0 (0·5, 3·5)	4·0 (2·9, 5·2)	7·8 (5·6, 10·3)	8·4 (7·2, 9·8)	8·1 (7·0, 9·2)	9·0 (7·7, 10·4)	10·0 (9·1, 11·0)	11·3 (10·1, 12·5)	13·4 (12·1, 14·8)	15·9 (14·1, 17·6)
25–34	0·4 (0·1, 0·7)	1·5 (0·8, 2·3)	5·8 (4·7, 7·0)	10·3 (8·8, 11·9)	12·1 (10·1, 14·0)	10·4 (8·7, 12·0)	11·4 (10·2, 12·7)	13·3 (12·3, 14·3)	13·7 (12·9, 14·6)	15·6 (14·7, 16·4)	17·3 (16·2, 18·3)
35–44	0·0 (0·0, 0·0)	1·0 (0·4, 1·5)	4·3 (3·1, 5·5)	8·8 (7·4, 10·2)	12·5 (10·8, 14·1)	11·1 (9·5, 12·8)	13·7 (12·6, 14·8)	14·4 (13·4, 15·3)	14·2 (13·4, 15·1)	14·2 (13·2, 15·1)	15·0 (13·9, 16·1)
45–54	0·0 (0·0, 0·0)	0·5 (0·2, 0·8)	2·8 (1·9, 4·0)	8·2 (7·2, 9·6)	10·2 (8·9, 11·8)	10·6 (9·1, 12·3)	13·5 (12·6, 14·5)	13·3 (12·4, 14·2)	12·9 (12·0, 13·8)	14·3 (13·3, 15·3)	15·5 (14·5, 16·6)
55–69	0·1 (0·0, 0·2)	0·4 (0·1, 0·6)	1·3 (0·8, 1·8)	4·3 (3·6, 5·2)	6·5 (5·6, 7·7)	5·3 (4·4, 6·4)	9·2 (8·5, 9·8)	9·1 (8·5, 9·8)	9·2 (8·5, 9·9)	9·6 (9·0, 10·3)	10·7 (9·9, 11·5)
Cigarette smoking											
Never	0·0 (0·0, 0·1)	0·2 (0·1, 0·3)	0·6 (0·4, 0·9)	0·2 (0·1, 0·3)	0·3 (0·1, 0·4)	0·3 (0·2, 0·5)	0·5 (0·4, 0·7)	1·1 (1·0, 1·3)	0·9 (0·8, 1·1)	1·3 (1·1, 1·5)	1·3 (1·1, 1·6)
Former	0·2 (0·1, 0·5)	0·5 (0·2, 1·0)	3·1 (2·1, 4·1)	8·4 (7·3, 9·6)	10·1 (8·9, 11·4)	8·9 (7·7, 10·3)	14·7 (13·8, 15·7)	15·5 (14·6, 16·3)	15·1 (14·5, 16·0)	16·3 (15·5, 17·1)	17·5 (16·6, 18·4)
Current	1·0 (0·5, 1·6)	3·6 (2·5, 4·8)	16·1 (13·8, 18·5)	27·4 (25·3, 29·7)	40·9 (38·2, 43·7)	34·9 (32·3, 37·1)	35·7 (34·2, 37·3)	38·5 (37·3, 39·9)	40·8 (39·5, 42·3)	41·8 (40·5, 43·1)	44·5 (43·0, 46·2)

Values are unweighted percentages with 95% confidence intervals in parentheses. Current HTP use was defined as use of at least one HTP product on at least 1 day in the past 30 days and comprised HTP-only and Dual use. No survey weights were applied. Confidence intervals were estimated using respondent-level cluster bootstrap resampling. CI, confidence interval; HTP, heated tobacco product.

S2. Longitudinal analyses

S2.1 Panel follow-up and transition-eligible sample

Annual analytic sample size, previous-wave follow-up status, and current-wave sample composition are summarised in Supplementary Table S5, Panel A. Previous-wave retention ranged from 62·5% to 75·6% across the 2016–2025 waves. Across origin-year observations from 2015–2024, 93,540 contributed a valid next-year transition pair, 44,568 were not observed in the next wave while remaining age-eligible, and 2,111 aged out of the analytic age range (Panel B). Participants' sex and age distributions by total number of survey waves contributed are shown in Panel C. Panel D summarises maximum consecutive-wave participation, showing progressive attenuation with sustained follow-up: 60·4% of respondents contributed at least two consecutive waves, decreasing to 38·6%, 24·3%, and 14·1% for at least three, four, and five consecutive waves, respectively.

S2.2 Knot selection in longitudinal analysis

Candidate one-knot years from 2017 to 2023 were compared using a parsimonious multinomial 1-year transition model without from-by-time interaction terms. 2018 provided the best fit, with the lowest BIC among all candidate knot years (BIC 90,730; Table S6). Although knot selection was based on the parsimonious model, the main longitudinal analyses subsequently used the richer model with from-by-time interaction terms. Relative to 2018, the next best candidate was 2017 (ΔBIC 41), whereas ΔBIC values for later knot years ranged from 114 to 228. These results indicate substantially worse fit for all alternative knot years and support selection of 2018 as the common change point for the longitudinal analyses.

S2.3 Full transition results

Full annual model-based, sex- and age-standardised 1-year transition probabilities across all five tobacco-use states are shown in Figure S1, and period-specific mean transition matrices for 2016–2018 and 2019–2025 are shown in Table S7. These supplementary results correspond to the selected 3×3 panels presented in Figure 2 of the main text. Across the full 5-state system, the largest probabilities generally reflected persistence within the same state, particularly for Never, Non-current, Cigarette-only, HTP-only, and Dual use. Over time, persistence of Cigarette-only declined, while transition from Cigarette-only to Non-current increased. By contrast, transition from Cigarette-only to HTP-only remained low throughout follow-up, and transition from Cigarette-only to Dual use rose around 2018 but did not continue to accelerate thereafter. Among HTP-only and Dual use respondents, persistence increased after 2018, whereas transition to Non-current use decreased. Estimates for transitions from HTP-only use in earlier years were less precise, as reflected in the wider confidence intervals.

S2.4 Detailed decomposition results

Detailed decomposition results are shown in Figure S2 and Table S8. Figure S2 presents annual route-specific contributions to change in HTP prevalence across all 10 transition routes, together with the annual net change in

reconstructed HTP prevalence. Table S8 summarizes period-specific mean annual contributions and slopes for broader aggregates, including Entry into HTP use, Uptake from Never/Non-current groups, switching from Cigarette-only to HTP, Exit from HTP use, and their component routes.

Across the full 10-route decomposition, positive contributions before 2018 were driven mainly by transitions from Cigarette-only to Dual use and, to a lesser extent, Cigarette-only to HTP-only use. After 2018, contributions from uptake among Never/Non-current groups increased, particularly transitions from Non-current use to Dual use and Non-current use to HTP-only use. At the same time, negative contributions also increased, especially through Dual use to Non-current use and HTP-only use to Non-current use. Despite these larger negative contributions, the annual net change in reconstructed HTP prevalence remained positive throughout 2016–2025. These supplementary results are consistent with the 4-category decomposition presented in Figure 3 of the main text and show in greater detail how the balance of contributing routes shifted over time.

Table S5. Analytic sample retention, next-wave follow-up status, and survey participation history in the Japan Society and New Tobacco Internet Survey, 2015–2025

Panel A. Annual analytic sample size, previous-wave follow-up status, and current-wave sample composition

Survey year	Total analytic respondents, n	Retained within analytic age range, n (%)	Not observed in current wave while age-eligible, n (%)	Aged out of analytic age range, n (%)	Returning after a gap, n	First-time JASTIS participants, n
2015	7,962	—	—	—	—	7,962
2016	5,774	5,256 (66·0)	2,661 (33·4)	45 (0·6)	0	518
2017	5,864	4,161 (72·1)	1,533 (26·6)	80 (1·4)	279	1,424
2018	8,595	4,436 (75·6)	1,331 (22·7)	97 (1·7)	437	3,722
2019	8,313	6,437 (74·9)	1,999 (23·3)	159 (1·8)	361	1,515
2020	8,033	6,026 (72·5)	2,137 (25·7)	150 (1·8)	512	1,495
2021	19,393	5,024 (62·5)	2,896 (36·1)	113 (1·4)	618	13,751
2022	25,481	13,334 (68·8)	5,736 (29·6)	323 (1·7)	947	11,200
2023	26,421	16,230 (63·7)	8,871 (34·8)	380 (1·5)	1,518	8,673
2024	24,383	17,270 (65·4)	8,779 (33·2)	372 (1·4)	2,368	4,745
2025	21,364	15,366 (63·0)	8,625 (35·4)	392 (1·6)	2,252	3,746

Annual analytic samples were restricted to respondents aged 18–69 years. Retained within the analytic age range denotes respondents in the preceding year's analytic sample who were observed in the current year and remained aged 18–69 years. Not observed in the current wave while age-eligible denotes respondents in the preceding year's analytic sample who remained aged 18–69 years but were not observed in the current year. Aged out of the analytic age range denotes respondents aged 18–69 years in the preceding year who would have been aged 70 years or older

in the current year, regardless of whether they participated in that wave. Returning after a gap denotes respondents observed in the current year who had participated in JASTIS before the preceding year but were not observed in the preceding year. First-time JASTIS participants were respondents first observed in JASTIS in the current year. Retained, not-observed, and aged-out respondents form mutually exclusive categories, and their percentages were calculated using the preceding year's analytic sample as the denominator. Values for 2015 are not applicable because no preceding wave was available. JASTIS eligibility was limited to respondents aged 69 years or younger through 2019 and subsequently expanded to include older adults; the present analyses were consistently restricted to respondents aged 18–69 years.

Panel B. Characteristics of origin-year observations by next-wave follow-up status

	Contributed a valid next-year transition pair, n (%)	Not observed in next wave while age-eligible, n (%)	Aged out of analytic age range before next wave, n (%)
Overall	93,540 (100·0)	44,568 (100·0)	2,111 (100·0)
Sex			
Man	48,349 (51·7)	20,780 (46·6)	1,049 (49·7)
Woman	45,191 (48·3)	23,788 (53·4)	1,062 (50·3)
Age, years			
18-24	7,882 (8·4)	9,180 (20·6)	0 (0·0)
25-34	16,103 (17·2)	10,864 (24·4)	0 (0·0)
35-44	19,582 (20·9)	8,310 (18·6)	0 (0·0)
45-54	21,546 (23·0)	7,458 (16·7)	0 (0·0)
55-69	28,427 (30·4)	8,756 (19·6)	2,111 (100·0)
Tobacco-use state in the origin year			
Never	48,784 (52·2)	25,165 (56·5)	972 (46·0)
Non-current	23,972 (25·6)	10,038 (22·5)	762 (36·1)
Cigarette-only	11,552 (12·3)	4,636 (10·4)	248 (11·7)
HTP-only	3,582 (3·8)	1,781 (4·0)	45 (2·1)
Dual	5,650 (6·0)	2,948 (6·6)	84 (4·0)

Origin-year observations were restricted to 2015–2024 because observations from 2025 had no subsequent survey wave in which to contribute a next-year transition pair. The three follow-up categories were mutually exclusive. A valid next-year transition pair required observations in two consecutive survey years, with the respondent aged 18–69 years and having a valid tobacco-use state in both years. Not observed in the next wave while age-eligible denotes origin-year observations for respondents who would have remained aged 18–69 years but were not observed in the subsequent wave. Aged out of the analytic age range denotes origin-year observations for respondents who would have been aged 70 years or older in the subsequent year, regardless of whether they participated in that wave. Respondents could contribute more than one origin-year observation. Age and tobacco-use state were measured in

the origin year. Values are unweighted counts and column percentages. HTP, heated tobacco product.

Panel C. Sex and age distribution of participants by total number of survey waves contributed

Number of survey waves contributed	Unique participants, n	Sex		Age at first observed wave				
		Men, n (%)	Women, n (%)	18-24, n (%)	25-34, n (%)	35-44, n (%)	45-54, n (%)	55-69, n (%)
Overall	58,751	31,833 (54·2)	26,918 (45·8)	11,926 (20·3)	13,222 (22·5)	11,403 (19·4)	9,630 (16·4)	12,570 (21·4)
1	20871	9,352 (44·8)	11,519 (55·2)	6,383 (30·6)	5,650 (27·1)	3,331 (16·0)	2,086 (10·0)	3,421 (16·4)
2	12366	5,518 (44·6)	6,848 (55·4)	2,474 (20·0)	3,257 (26·3)	2,348 (19·0)	1,810 (14·6)	2,477 (20·0)
3	9112	4,302 (47·2)	4,810 (52·8)	1,313 (14·4)	1,943 (21·3)	2,159 (23·7)	1,619 (17·8)	2,078 (22·8)
4	6855	3,444 (50·2)	3,411 (49·8)	866 (12·6)	1,071 (15·6)	1,470 (21·4)	1,556 (22·7)	1,892 (27·6)
≥5	9547	5,217 (54·6)	4,330 (45·4)	890 (9·3)	1,301 (13·6)	2,095 (21·9)	2,559 (26·8)	2,702 (28·3)

Age was defined at the participant's first observed survey wave. The total number of waves contributed was not necessarily consecutive. Percentages for sex and age are row percentages.

Panel D. Distribution of participants by maximum consecutive-wave participation

Consecutive waves contributed	Unique participants, n (%)
≥2 consecutive waves	35,479 (60·4%)
≥3 consecutive waves	22,657 (38·6%)
≥4 consecutive waves	14,250 (24·3%)
≥5 consecutive waves	8,264 (14·1%)

Percentages were calculated among all 58,751 unique respondents. Consecutive-wave participation was defined according to the maximum number of consecutive annual survey waves contributed by each respondent.

Table S6. Candidate knot years for longitudinal transition model

Knot year	BIC	ΔBIC	Selected
2018	90,730	0	Yes
2017	90,771	41	No
2019	90,844	114	No
2020	90,934	204	No
2021	90,949	219	No
2022	90,950	220	No
2023	90,958	228	No

Lower BIC indicates better fit. ΔBIC is shown relative to the best-fitting model (knot year 2018). Candidate knot years were evaluated using a parsimonious multinomial 1-year transition model without from-by-time interaction terms.

Table S7. Period-specific mean model-based, sex- and age-standardised 1-year transition probabilities between five tobacco-use states in Japan, 2016–2018 and 2019–2025

(a) 2016–2018

		To				
		Never	Non-current	Cigarette-only	HTP-only	Dual
From	Never	94·4 (94·0, 94·8)	2·8 (2·5, 3·0)	2·2 (1·9, 2·5)	0·2 (0·2, 0·3)	0·4 (0·3, 0·5)
	Non-current	–	93·9 (93·1, 94·6)	4·7 (4·1, 5·4)	0·5 (0·3, 0·7)	0·9 (0·7, 1·2)
	Cigarette-only	–	13·5 (12·2, 14·8)	76·8 (75·3, 78·4)	2·1 (1·6, 2·6)	7·6 (6·7, 8·5)
	HTP-only	–	36·4 (6·0, 61·0)	2·0 (1·0, 3·2)	43·1 (25·4, 69·8)	18·6 (4·1, 40·9)
	Dual	–	26·0 (8·9, 39·8)	12·9 (6·2, 21·5)	16·2 (6·1, 33·3)	44·9 (32·4, 58·8)

(b) 2019–2025

		To				
		Never	Non-current	Cigarette-only	HTP-only	Dual
From	Never	92·3 (92·0, 92·6)	5·1 (4·8, 5·3)	1·3 (1·2, 1·4)	0·5 (0·5, 0·6)	0·8 (0·7, 0·8)
	Non-current	–	89·1 (88·6, 89·6)	7·3 (6·9, 7·7)	1·1 (0·9, 1·2)	2·5 (2·3, 2·7)
	Cigarette-only	–	22·0 (20·9, 22·9)	64·4 (63·3, 65·5)	2·7 (2·4, 3·0)	11·0 (10·3, 11·7)
	HTP-only	–	15·5 (14·0, 17·1)	2·8 (2·2, 3·6)	67·9 (65·8, 70·0)	13·8 (12·5, 15·3)
	Dual	–	15·3 (14·1, 16·5)	14·5 (13·3, 15·7)	13·2 (12·2, 14·3)	57·0 (55·2, 58·7)

Values are percentages with 95% confidence intervals in parentheses. Rows indicate origin states and columns indicate destination states. Probabilities were standardised to the pooled sex-by-age distribution of the transition-eligible sample. A dash (–) indicates structurally impossible transitions to Never because Never was treated as a cumulative state. Row totals may not equal 100% because of rounding.

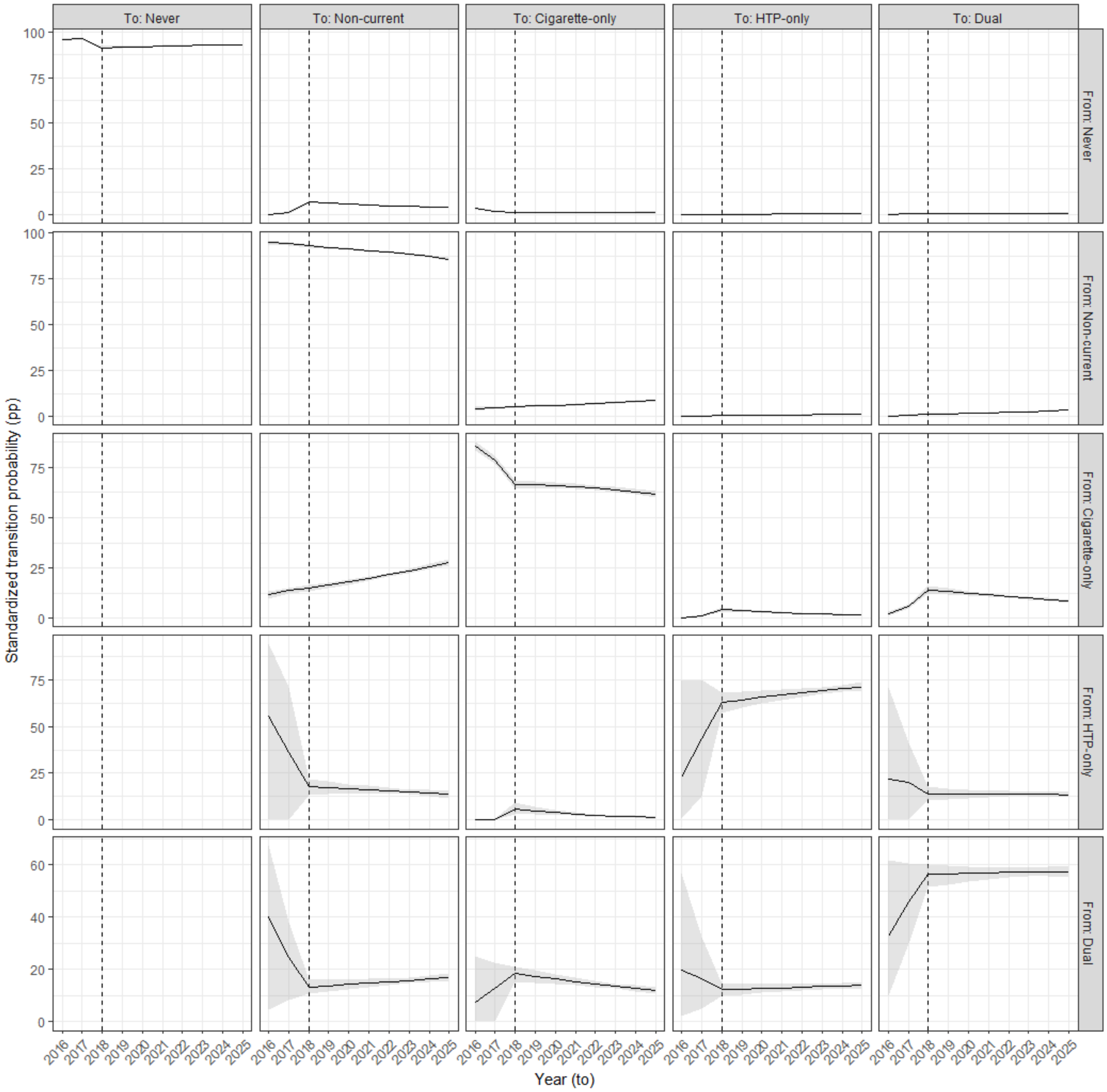

Figure S1. Full annual model-based, sex- and age-standardised 1-year transition probabilities between five tobacco-use states in Japan, 2016–2025. Rows indicate origin states and columns indicate destination states. Grey bands indicate 95% cluster bootstrap confidence intervals. The dashed vertical line marks 2018, the selected common change point used in period-specific summaries.

Table S8. Period-specific mean annual contributions and slopes from the Markov decomposition of annual change in heated tobacco product prevalence

	2016–2018		2019–2025	
	Mean annual contribution, pp (95% CI)	Slope, pp/year (95% CI)	Mean annual contribution, pp (95% CI)	Slope, pp/year (95% CI)
Entry to HTP use	2·16 (1·96, 2·35)	**1·38 (1·20, 1·60)**	3·48 (3·33, 3·62)	**0·16 (0·10, 0·21)**
Uptake (Never/Non-current → HTP)	0·73 (0·62, 0·85)	**0·22 (0·12, 0·33)**	1·92 (1·81, 2·03)	**0·28 (0·23, 0·33)**
Never → HTP-only	0·14 (0·10, 0·20)	0·02 (−0·03, 0·07)	0·21 (0·19, 0·24)	**0·02 (0·001, 0·03)**
Never → Dual	0·25 (0·19, 0·32)	0·07 (−0·003, 0·13)	0·31 (0·28, 0·34)	−0·002 (−0·02, 0·01)
Non-current → HTP-only	0·12 (0·07, 0·16)	0·02 (−0·03, 0·07)	0·42 (0·36, 0·47)	**0·08 (0·06, 0·11)**
Non-current → Dual	0·22 (0·16, 0·28)	**0·12 (0·06, 0·17)**	0·98 (0·89, 1·06)	**0·18 (0·14, 0·22)**
Switch from Cigarette-only to HTP	1·43 (1·28, 1·57)	**1·16 (1·00, 1·35)**	1·56 (1·47, 1·66)	**−0·12 (−0·16, −0·09)**
Cigarette-only → HTP-only	0·31 (0·24, 0·38)	**0·29 (0·21, 0·39)**	0·31 (0·27, 0·35)	**−0·04 (−0·06, −0·02)**
Cigarette-only → Dual	1·12 (0·99, 1·25)	**0·87 (0·73, 1·03)**	1·26 (1·18, 1·34)	**−0·08 (−0·12, −0·05)**
Exit from HTP use	−0·39 (−0·46, −0·31)	**−0·33 (−0·41, −0·26)**	−2·30 (−2·43, −2·17)	**−0·21 (−0·26, −0·16)**
Exit to Non-current	−0·23 (−0·30, −0·16)	**−0·15 (−0·21, −0·10)**	−1·41 (−1·50, −1·32)	**−0·18 (−0·22, −0·14)**
Dual → Non-current	−0·15 (−0·20, −0·10)	**−0·10 (−0·14, −0·06)**	−0·88 (−0·96, −0·81)	**−0·11 (−0·14, −0·08)**
HTP-only → Non-current	−0·09 (−0·13, −0·04)	**−0·05 (−0·09, −0·03)**	−0·52 (−0·58, −0·48)	**−0·07 (−0·09, −0·05)**
Switch back to Cigarette-only	−0·15 (−0·20, −0·11)	**−0·18 (−0·22, −0·14)**	−0·90 (−0·98, −0·82)	−0·03 (−0·05, 0·001)
Dual → Cigarette-only	−0·14 (−0·19, −0·10)	**−0·16 (−0·20, −0·12)**	−0·81 (−0·89, −0·74)	**−0·03 (−0·05, −0·002)**
HTP-only → Cigarette-only	−0·01 (−0·02, −0·01)	**−0·02 (−0·04, −0·01)**	−0·09 (−0·11, −0·07)	0·001 (−0·01, 0·01)

Values are mean annual contributions (percentage points) or slopes in annual contributions (percentage points per year), with 95% confidence intervals in parentheses. Positive values indicate contributions to increasing HTP prevalence and negative values indicate contributions to decreasing HTP prevalence. **Bold** indicates 95% confidence intervals excluding 0. Period summaries were based on the aligned standardised transition sample.

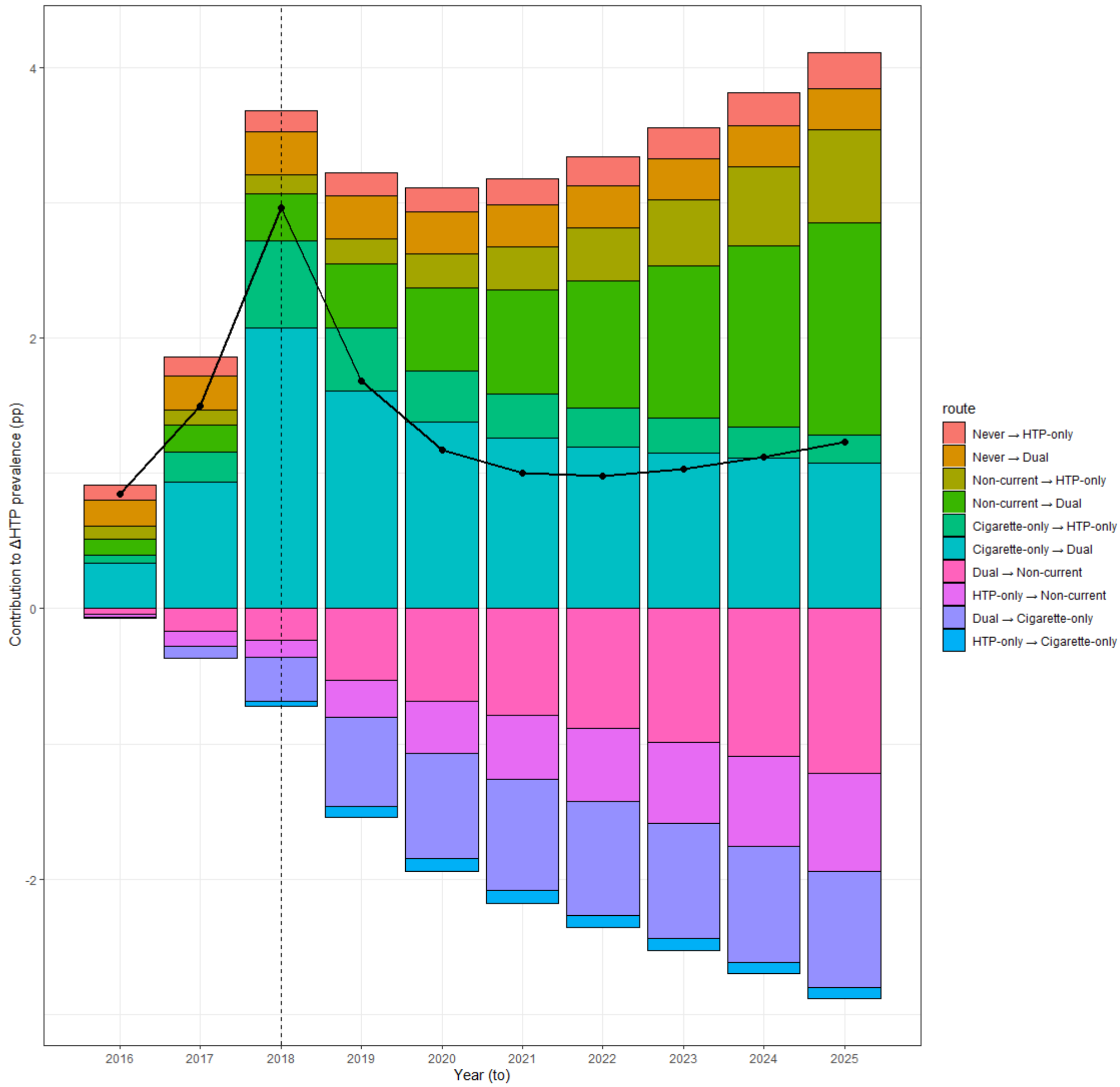

Figure S2. Annual route-specific contributions to change in heated tobacco product prevalence in Japan, 2016–2025, across 10 transition routes. Bars show annual contributions of each transition route to the change in reconstructed HTP prevalence, expressed in percentage points. Positive values indicate contributions to increasing HTP prevalence and negative values indicate contributions to decreasing HTP prevalence. The black line indicates the annual net change in reconstructed HTP prevalence in the aligned standardised transition sample. The dashed vertical line marks 2018, the selected common change point used in period-specific summaries.

S3. Sensitivity analyses

S3.1 Simpler transition model: model fit and reconstruction diagnostics

To assess structural sensitivity, we compared the main multinomial transition model including from×time interaction terms with a simpler model assuming common time trends across origin states. As shown in Table S9, the main model showed better fit and slightly better reconstruction of aligned HTP prevalence than the simpler model. The main model had a lower BIC (90,354 vs 90,729) and lower discrepancy between reconstructed and observed aligned HTP prevalence, including lower mean absolute HTP difference (0·76 vs 0·78 percentage points), lower median absolute HTP difference (0.55 vs 0.66 percentage points), and lower maximum absolute HTP difference (2·24 vs 2·42 percentage points). It also showed lower mean RMSE (0·042 vs 0·043) and mean MAD (0·029 vs 0·030) for the full five-state distribution. These results supported retention of the from×time model as the main longitudinal model.

S3.2 Sensitivity analysis using the simpler transition model

Sensitivity analyses based on the simpler transition model without from×time interaction terms are shown in Tables S10 and S11. Overall, the simpler model produced broadly similar substantive conclusions to the main model. Period-specific transition probabilities under the simpler model continued to show high persistence of HTP-only and Dual use after 2018 and limited transition from Cigarette-only to HTP-only use (Table S10). Likewise, aggregated decomposition summaries under the simpler model supported the same overall interpretation as the main model: positive net change in reconstructed HTP prevalence was maintained by both switching from Cigarette-only to HTP use and uptake from Never/Non-current groups, despite larger negative contributions from exit routes after 2018 (Table S11).

S3.3 Additional sensitivity analyses

Additional adjustment for time in sample produced decomposition estimates that were materially similar to those from the main analysis (Table S12). During 2019–2025, uptake from Never/Non-current states continued to increase (slope, 0·27 percentage points/year; 95% CI, 0·21 to 0·33), whereas the contribution from switching from Cigarette-only to HTP use declined over time (−0·10 percentage points/year; 95% CI, −0·15 to −0·06). Findings were also materially unchanged when the analyses were restricted to respondents aged 25–69 years (Table S13), with corresponding post-2018 slopes of 0·27 percentage points/year (0·23 to 0·32) for uptake from Never/Non-current states and −0·14 percentage points/year (−0·17 to −0·10) for switching from Cigarette-only to HTP use.

Table S9. Comparison of model fit and reconstruction diagnostics between the main and simpler longitudinal transition models

Model	BIC	Mean absolute HTP difference, pp	Median absolute HTP difference, pp	Maximum absolute HTP difference, pp	Mean RMSE	Mean MAD
Simpler model (base)	90,728·93	0·78	0·66	2·42	0·043	0·030
Main model (from×time)	90,353·89	0·76	0·55	2·24	0·042	0·029

Lower BIC, absolute HTP difference, RMSE, and MAD indicate better fit. Absolute HTP difference was defined as the absolute difference between reconstructed HTP prevalence and observed aligned HTP prevalence for each year. RMSE and MAD were calculated for the full five-state distribution. Diagnostics were summarized across annual values from 2016 to 2025.

Table S10. Period-specific mean model-based, sex- and age-standardised 1-year transition probabilities between five tobacco-use states in Japan under the simpler transition model, 2016–2018 and 2019–2025

(a) 2016–2018

		To: Never	Non-current	Cigarette-only	HTP-only	Dual
From	Never	94·3 (93·8, 94·7)	3·6 (3·4, 3·9)	1·4 (1·2, 1·6)	0·3 (0·2, 0·3)	0·4 (0·4, 0·5)
	Non-current	–	88·6 (87·9, 89·2)	9·1 (8·6, 9·7)	0·7 (0·5, 0·8)	1·6 (1·4, 1·8)
	Cigarette-only	–	19·7 (18·6, 20·9)	72·8 (71·5, 74·2)	1·4 (1·1, 1·6)	6·1 (5·5, 6·7)
	HTP-only	–	24·0 (20·5, 28·3)	5·0 (3·6, 6·3)	58·8 (52·6, 64·1)	12·2 (10·4, 14·6)
	Dual	–	21·1 (19·0, 23·2)	24·8 (22·6, 27·0)	9·9 (8·0, 12·0)	44·2 (40·6, 48·0)

(b) 2019–2025

		To: Never	Non-current	Cigarette-only	HTP-only	Dual
From	Never	92·4 (92·1, 92·6)	4·7 (4·5, 5·0)	1·5 (1·4, 1·7)	0·6 (0·5, 0·7)	0·8 (0·7, 0·9)
	Non-current	–	89·3 (88·8, 89·8)	6·9 (6·6, 7·3)	1·1 (1·0, 1·3)	2·6 (2·4, 2·9)
	Cigarette-only	–	22·2 (21·3, 23·3)	64·6 (63·3, 65·8)	2·5 (2·2, 2·8)	10·6 (10·0, 11·3)
	HTP-only	–	15·6 (14·3, 17·1)	2·3 (1·8, 2·8)	68·0 (66·2, 69·9)	14·0 (12·8, 15·2)
	Dual	–	16·1 (15·0, 17·2)	13·8 (12·8, 14·7)	13·1 (12·1, 14·1)	57·0 (55·4, 58·7)

Values are percentages with 95% confidence intervals in parentheses. Rows indicate origin states and columns indicate destination states. Probabilities were standardised to the pooled sex-by-age distribution of the transition-eligible sample. A dash (–) indicates structurally impossible transitions to Never because Never was treated as a

cumulative state. Row totals may not equal 100% because of rounding.

Table S11. Period-specific mean annual contributions and slopes from the Markov decomposition of annual change in heated tobacco product prevalence in Japan under the simpler transition model

	2016–2018		2019–2025	
	Mean annual contribution, pp (95% CI)	Slope, pp/year (95% CI)	Mean annual contribution, pp (95% CI)	Slope, pp/year (95% CI)
Entry to HTP use	2·06 (1·90, 2·22)	**1·23 (1·06, 1·40)**	3·51 (3·38, 3·65)	**0·11 (0·07, 0·16)**
Uptake (Never/Non-current → HTP)	0·97 (0·89, 1·06)	**0·59 (0·50, 0·67)**	2·00 (1·90, 2·11)	**0·08 (0·05, 0·11)**
Switch from Cigarette-only to HTP	1·09 (1·00, 1·18)	**0·64 (0·53, 0·75)**	1·50 (1·42, 1·59)	**0·03 (0·01, 0·05)**
Exit from HTP use	−0·39 (−0·45, −0·35)	**−0·31 (−0·37, −0·26)**	−2·31 (−2·42, −2·19)	**−0·23 (−0·25, −0·22)**
Exit to Non-current	−0·23 (−0·27, −0·20)	**−0·20 (−0·24, −0·17)**	−1·44 (−1·53, −1·35)	**−0·15 (−0·16, −0·13)**
Switch back to Cigarette-only	−0·16 (−0·19, −0·14)	**−0·11 (−0·13, −0·09)**	−0·87 (−0·93, −0·80)	**−0·09 (−0·10, −0·08)**

Values are mean annual contributions (percentage points) or slopes in annual contributions (percentage points per year), with 95% confidence intervals in parentheses. Positive values indicate contributions to increasing HTP prevalence and negative values indicate contributions to decreasing HTP prevalence. Bold indicates 95% confidence intervals excluding 0. Period summaries were based on the aligned standardised transition sample.

Table S12. Period-specific mean annual contributions and slopes from the Markov decomposition of annual change in heated tobacco product prevalence under additional adjustment for time in sample

	2016–2018		2019–2025	
	Mean annual contribution, pp (95% CI)	Slope, pp/year (95% CI)	Mean annual contribution, pp (95% CI)	Slope, pp/year (95% CI)
Entry to HTP use	2·07 (1·81, 2·40)	**1·34 (1·05, 1·61)**	3·48 (3·25, 3·66)	**0·16 (0·09, 0·24)**
Uptake (Never/Non-current → HTP)	0·70 (0·54, 0·92)	**0·27 (0·07, 0·43)**	1·95 (1·75, 2·10)	**0·27 (0·21, 0·33)**
Switch from Cigarette-only to HTP	1·37 (1·17, 1·55)	**1·07 (0·85, 1·29)**	1·52 (1·42, 1·65)	**−0·10 (−0·15, −0·06)**
Exit from HTP use	−0·37 (−0·48, −0·27)	**−0·31 (−0·43, −0·20)**	−2·30 (−2·47, −2·08)	**−0·22 (−0·27, −0·17)**
Exit to Non-current	−0·21 (−0·31, −0·14)	**−0·14 (−0·23, −0·06)**	−1·42 (−1·52, −1·28)	**−0·18 (−0·23, −0·15)**
Switch back to Cigarette-only	−0·16 (−0·22, −0·09)	**−0·18 (−0·24, −0·12)**	−0·88 (−0·97, −0·80)	−0·03 (−0·06, 0·01)

Time in sample was defined as the cumulative number of JASTIS survey waves contributed up to and including the origin wave and was categorised as 1, 2, 3, 4, or ≥5 waves. The multinomial transition model additionally included time-in-sample category while retaining the segmented time structure and origin-state-by-time interactions used in the main model. Predicted transition probabilities were averaged over the pooled distribution of time in sample

within sex-by-age strata and then standardised to the pooled sex-by-age distribution of the transition-eligible sample. Values are mean annual contributions in percentage points or slopes in annual contributions in percentage points per year, with 95% confidence intervals in parentheses. Positive values indicate contributions to increasing HTP prevalence, and negative values indicate contributions to decreasing HTP prevalence. Bold indicates 95% confidence intervals excluding 0.

Table S13. Period-specific mean annual contributions and slopes from the Markov decomposition of annual change in heated tobacco product prevalence among respondents aged 25–69 years

	2016–2018		2019–2025	
	Mean annual contribution, pp (95% CI)	Slope, pp/year (95% CI)	Mean annual contribution, pp (95% CI)	Slope, pp/year (95% CI)
Entry to HTP use	2·06 (1·88, 2·26)	**1·33 (1·12, 1·54)**	3·25 (3·12, 3·38)	**0·14 (0·08, 0·20)**
Uptake (Never/Non-current → HTP)	0·62 (0·51, 0·74)	**0·14 (0·02, 0·25)**	1·69 (1·59, 1·81)	**0·27 (0·23, 0·32)**
Switch from Cigarette-only to HTP	1·44 (1·29, 1·59)	**1·20 (1·02, 1·37)**	1·55 (1·46, 1·65)	**−0·14 (−0·17, −0·10)**
Exit from HTP use	−0·35 (−0·42, −0·27)	**−0·30 (−0·38, −0·24)**	−2·12 (−2·24, −2·00)	**−0·20 (−0·24, −0·15)**
Exit to Non-current	−0·19 (−0·25, −0·13)	**−0·13 (−0·18, −0·09)**	−1·24 (−1·33, −1·16)	**−0·18 (−0·21, −0·14)**
Switch back to Cigarette-only	−0·15 (−0·20, −0·11)	**−0·18 (−0·22, −0·14)**	−0·88 (−0·96, −0·81)	−0·02 (−0·05, 0·01)

The sensitivity analysis was restricted to consecutive-year transition pairs in which respondents were aged 25–69 years in both the origin and destination years. Models and standardisation procedures otherwise followed those used in the main analysis. Values are mean annual contributions in percentage points or slopes in annual contributions in percentage points per year, with 95% confidence intervals in parentheses. Positive values indicate contributions to increasing HTP prevalence, and negative values indicate contributions to decreasing HTP prevalence. Bold indicates 95% confidence intervals excluding 0.
